# Histo-blood group antigen-binding specificities of human rotaviruses are associated with gastroenteritis but not with *in vitro* infection

**DOI:** 10.1038/s41598-018-31005-4

**Published:** 2018-08-28

**Authors:** Laure Barbé, Béatrice Le Moullac-Vaidye, Klara Echasserieau, Karine Bernardeau, Thomas Carton, Nicolai Bovin, Johan Nordgren, Lennart Svensson, Nathalie Ruvoën-Clouet, Jacques Le Pendu

**Affiliations:** 1 CRCINA, Inserm, Université d’Angers, Université de Nantes, Nantes, France; 2 Plateforme P2R « Production de protéines recombinantes », SFR Sante F. Bonamy-IRS-UN, Université de Nantes, INSERM, CNRS, CHU Nantes, Nantes, France; 3 Biofortis, Mérieux NutriSciences, Nantes, France; 4 Institute of Bioorganic Chemistry RAS, Moscow, Russia; 5 Division of Molecular Virology, Medical Faculty, University of Linköping, Linköping, Sweden; 6 Division of Infectious Diseases, Department of Medicine Solna, Karolinska Institute, Stockholm, Sweden; 7 Oniris, Ecole Nationale Vétérinaire, Agroalimentaire et de l’Alimentation, Nantes, France

## Abstract

Human strains of rotavirus A (RVAs) recognize fucosylated glycans belonging to histo-blood group antigens (HBGAs) through their spike protein VP8*. Lack of these ligands due to genetic polymorphisms is associated with resistance to gastroenteritis caused by P[8] genotype RVAs. With the aim to delineate the contribution of HBGAs in the process, we analyzed the glycan specificity of VP8* proteins from various P genotypes. Binding to saliva of VP8* from P[8] and P[4] genotypes required expression of both FUT2 and FUT3 enzymes, whilst binding of VP8* from the P[14] genotype required FUT2 and A enzymes. We further defined a glycan motif, GlcNAcβ3Galβ4GlcNAc, recognized by P[6] clinical strains. Conversion into Lewis antigens by the FUT3 enzyme impaired recognition, explaining their lower binding to saliva of Lewis positive phenotype. In addition, the presence of neutralizing antibodies was associated with the presence of the *FUT2* wild type allele in sera from young healthy adults. Nonetheless, *in vitro* infection of transformed cell lines was independent of HBGAs expression, indicating that HBGAs are not human RV receptors. The match between results from saliva-based binding assays and the epidemiological data indicates that the polymorphism of human HBGAs controls susceptibility to RVAs, although the exact mechanism remains unclear.

## Introduction

Rotavirus (RV) is the leading cause of severe acute gastroenteritis in infants and children less than 5 years of age. It is a non-enveloped virus belonging to the *Reoviridae* family. Its genome consists of eleven segments of double-stranded RNA (dsRNA) encoding six structural and five non-structural proteins^1^. Structural proteins (or viral proteins VP1 to VP7) constitute the three concentric protein layers of the icosahedral capsid. The VP6 protein, forming the intermediate layer, is used for strain classification and enables the serogroup definition^2^. Among the eight serogroups (A to H), only serogroups A, B and C are pathogenic for humans. However, serogroups B and C do not seem to be of epidemiological importance outside China^3^. Two other structural proteins are used for strain classification: VP7 (G - Glycoprotein) and VP4 (P - Protease Sensitive) found on the outmost surfaces of the virions^1^. Genes encoding these proteins enable the definition of G and P genotypes respectively. Thus, 27 G genotypes and 37 P genotypes have been described^4^. However, the majority of human cases is due to infections by G1P[8], G2P[4], G3P[8] and G9P[8] genotypes of RVA^5^. VP4 proteins form spicules which bind to molecules from the surface of enterocytes allowing entry of the virus^6^. Virus infectivity is activated by proteolytic cleavage of VP4 and thus of the outer layer of the capsid, generating the VP5* and VP8* proteins^7^. The VP8* protein, the globular head of the spike, is believed to be involved in the attachment of virions to host cells^8^ and the VP5* protein, the stalk of the spike, in the translocation of the double-shelled particles into the cytoplasm through conformational rearrangements and membrane fusion^6,9,10^.

Based on a sensitivity test to sialidase, cell attachment and entry of RVs have long been considered to be dependent or independent on the presence of sialic acids on cell surface glycoconjugates such as gangliosides^9^. Previous studies have shown that only a few P genotypes of animal strains are “sialidase-sensitive” whereas human strains and the majority of animal strains are “sialidase-insensitive”^11^. However, most sialidases cleave only terminal sialic acids on glycoconjugates, without affecting sub-terminal sialic acids and more recent studies concluded that the sialic acid-independent human strains may recognize sialic acids internally located in the glycan chain like on the ganglioside GM1a^12–14^. RVs may also interact with other cell surface molecules including integrins and the heat shock cognate protein 70 (hsc70)^14^.

**Figure 1:**
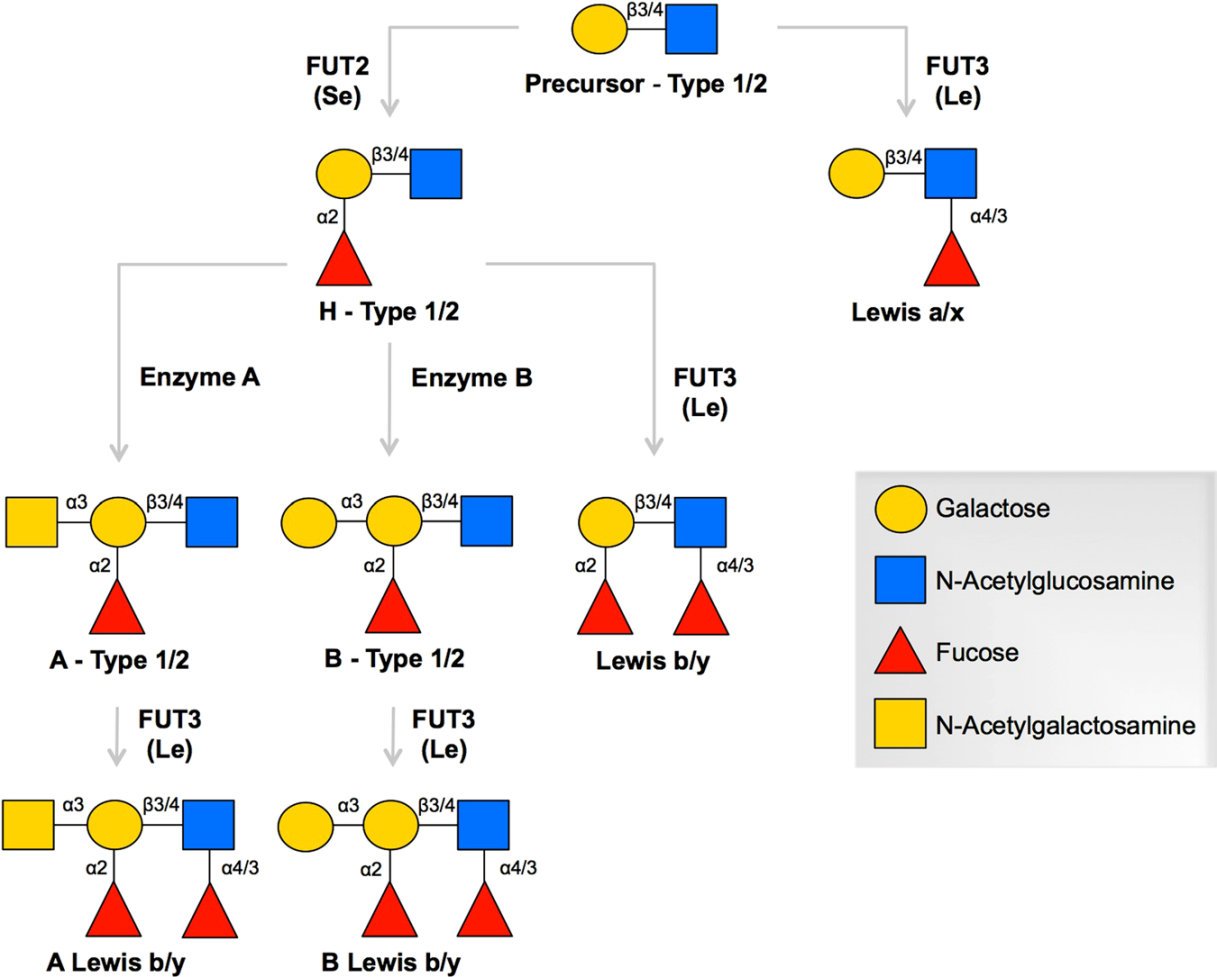
Biosynthesis pathway of HBGAs. HBGAs are synthesized by sequential addition of a monosaccharide to the terminal disaccharide of a precursor glycan. The synthesis pathways with type 1 and 2 precursors are considered here. Glycosidic linkages are separated by a forward slash to specify the pathway for different chains. The FUT2 and FUT3 fucosyltransferases drive the biosynthesis of secretor (Se) and Lewis (Le) antigens respectively by catalyzing the linkage-specific addition of α-fucose residues. Enzymes A and B catalyze the linkage-specific addition of N-acetylgalactosamine and galactose, respectively. Additions of glycans by enzymes A, B and FUT2 to the galactose residue of the precursor result in ABH HBGAs. Addition of α-fucose to the N-acetylglucosamine residue by FUT3 enzyme yields Lewis antigens (Lewis a/x, Lewis b/y, A Lewis b/y, B Lewis b/y).

Recent studies also suggested that the cell attachment protein VP8* can interact specifically with Histo-Blood Group Antigens (HBGAs), namely the ABH and Lewis antigens. These are complex carbohydrates found on the surface of many cell types, including epithelial intestinal cells and as free molecules in biologic fluids, such as saliva and milk^15,16^. The biosynthesis pathway of HBGAs (Fig. 1) starts with a disaccharide precursor followed by sequential additions of monosaccharides catalyzed by glycosyltransferases encoded by three major genes, the *ABO*, secretor (*FUT2*) and Lewis (*FUT3*) genes. Each comprises silent alleles, leading to null phenotypes in the homozygous state. An initial study showed a specific binding of recombinant VP8* proteins from clinical and cell culture-adapted strains with human saliva, milk or synthetic HBGA oligosaccharides^17^. Human strains of P[4] and P[8] genotypes showed a specific binding to H type 1 and Lewis b antigens whereas P[6] strains recognized H type 1 only. Further studies confirmed these results demonstrating binding of P[8] and P[4] VP8* proteins to saliva samples from secretor individuals^18–20^. Other studies also documented that P[9], P[14] and P[25] strains interact with the A antigen and that this interaction is involved in the infection *in vitro*^21–23^.

H type 1, Lewis b and A antigens have in common a fucose residue, α(1,2)-linked onto a galactose residue. This fucose is added by the α(1,2)-fucosyltransferase enzyme encoded by the *FUT2* gene, responsible for the “secretor” phenotype (SE). Lewis antigens also present a fucose residue, α(1,3/4)-linked onto a N-acetylglucosamine residue. It is added by the α(1,3/4)-fucosyltransferase enzyme encoded by the *FUT3* gene, responsible for the “Lewis positive” phenotype (Le^+^).

Mutations in *FUT2* or *FUT3* lead to a total absence or a severe decrease of the corresponding fucosyltransferase activity, ultimately causing the absence of α(1,2)- or α(1,3/4)-fucosylated antigens in mucous membranes and secretions. This determines the “non-secretor” (se) phenotype (or FUT2^−^) and the “Lewis negative” phenotype (or FUT3^−^). In a previous study, our team documented that the “non-secretor” phenotype was associated with resistance to severe gastroenteritis caused by P[8] human strains of RVA, which are predominant in Europe^24^. Thus, the results of the study suggested that the recognition of α(1,2)-fucosylated antigens by the VP8* protein could be essential for symptomatic infection by P[8] strains. Other epidemiological studies confirmed and completed these results^20,25–28^. Recent studies further underscored the importance of the secretor status by showing elevated RV specific antibodies in the serum and saliva of secretor individuals^20,29,30^. Only one study performed in Tunisia showed some discrepancy with these findings for P[8] strains^31^.

Currently, two live-attenuated vaccines are licensed and available in many countries. Since their introduction, the number of deaths associated with RV infection has decreased from 450,000 to about 200,000 in 2011^32,33^. The monovalent Rotarix^®^ vaccine (Glaxo-SmithKline) is derived from an attenuated human strain of G1P[8] genotype and the pentavalent RotaTeq^®^ vaccine (Merck) uses a bovine strain as genetic basis for five reassortants with human G1, G2, G3, G4 and P[8]^34^. According to recent studies, currently licensed vaccines are very effective (from 70 to more than 90% of protection) in high-income countries, but much less (20 to 30%) in low-income countries, which are the most affected^4^. Reasons for this lack of efficacy are not fully understood yet and probably multifactorial^35^. However, since both vaccines include a live-attenuated RV of P[8] genotype, the fact that Lewis negative children are resistant to P[8] strains could contribute to explain the reduced efficacy of vaccines in countries where the frequency of Lewis negative individuals is greater, such as Africa.

In light of these results, we make the hypothesis that RVA would first bind to fucosylated structures through their VP8* protein and secondly to sialic acids residues on gangliosides or to other cell surface receptors such as integrins or hsc70^14^, which would mediate virus entry. This hypothesis accounts for the fact that fucosylated structures are accessible in the glycocalyx thick layer, which length is around 500 nm on small intestine enterocytes, far more than gangliosides that extend no more than a few nanometers above the lipid bilayer^36^. With the aim to delineate the relative contribution of HBGAs in the infection process and to explore the consequences of HBGAs polymorphism on the virus transmission and efficacy of the available vaccines, we investigated the HBGA specificity of VP8* proteins from strains of various P genotypes, including the vaccine strains and the effect of HBGAs synthesis blockade on the *in vitro* infection of susceptible cells. We further analyzed the presence of neutralizing antibodies in sera from young healthy adults of known HBGA types.

## Results

### P[8] and P[4] VP8* proteins bind to difucosylated structures

**Figure 2:**
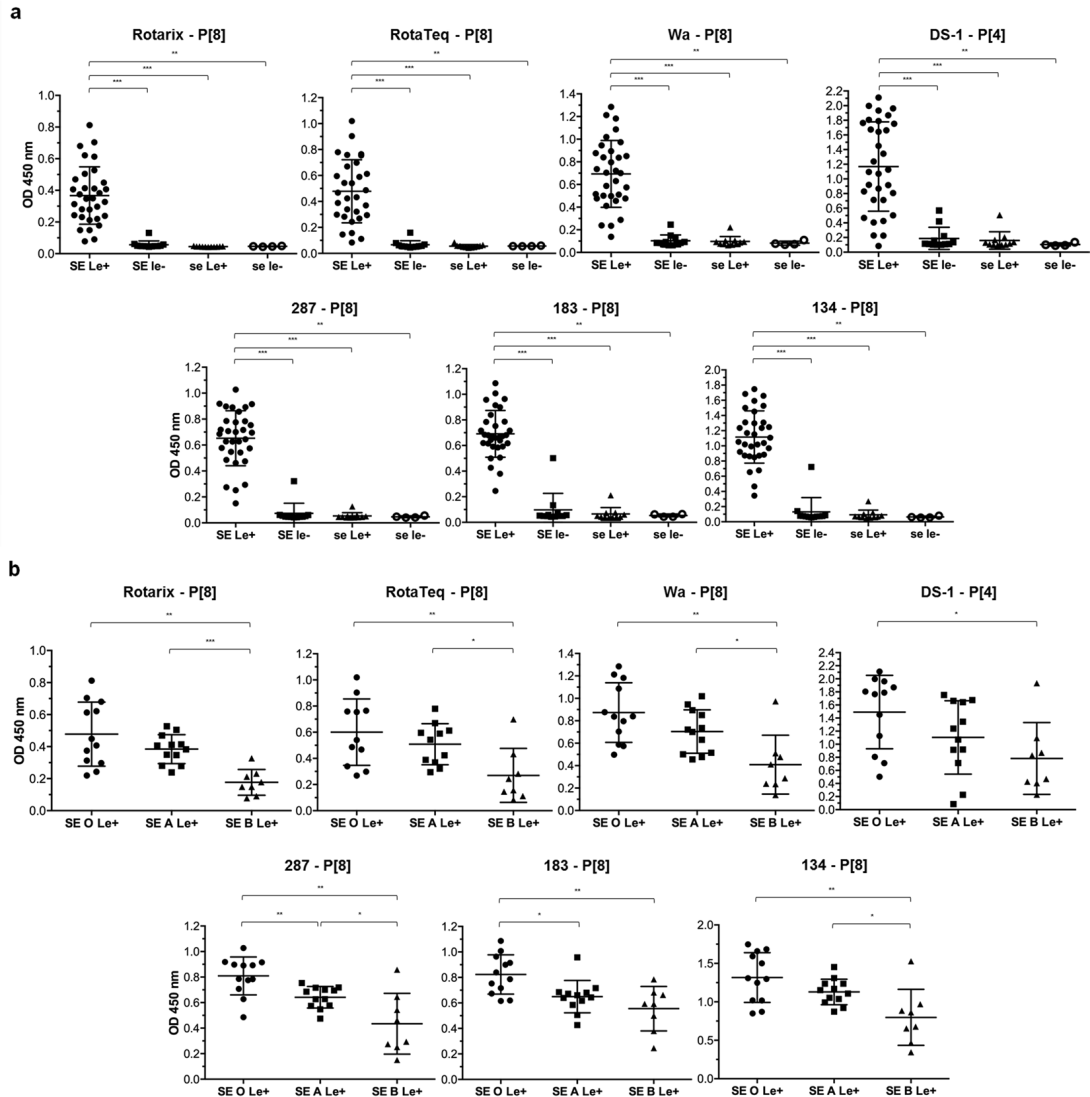
Binding of human strains VP8* to HBGAs measured by saliva-based binding assay. VP8* proteins from vaccine strains (Rotarix (P[8]) and RotaTeq (P[8])), cell culture-adapted strains (Wa (P[8]) and DS-1 (P[4])) and three P[8] clinical strains (287, 183 and 134) were tested for binding to a panel of previously well-defined A, B, O, secretor and Lewis types saliva samples (SE = Secretor (FUT2^+^); se = non-secretor (FUT2^−^); Le^+^ = Lewis positive (FUT3^+^); le^−^ = Lewis negative (FUT3^−^); O, A and B = Blood group O, A and B). **(a)** The binding profile of VP8* proteins to HBGAs is identical for all the strains tested with a binding to mucins of SE/Le^+^ phenotypes only (SE/Le^+^ n = 32, SE/le^−^ n = 12, se/Le^+^ n = 11 and se/le^−^ n = 4). **(b)** Within the SE/Le^+^ group, ABO phenotype has an effect with a binding to saliva samples from blood group O ≥ A > B (O n = 12, A n = 12 and B n = 8). Mann-Whitney test was used to compare groups (***p < 0.001, **p < 0.01, *p < 0.05).

We started by studying the binding of recombinant VP8* proteins from human strains to HBGAs by saliva-based binding assay since human saliva contains mucins that bear HBGAs, similar to those present at the surface of the small intestine^37^. VP8* proteins from vaccine strains (Rotarix P[8] and RotaTeq P[8]), cell culture-adapted strains (Wa P[8] and DS-1 P[4]) and three representative P[8] clinical strains (287, 183 and 134) were tested for binding to a panel of previously well-defined A, B, O, secretor and Lewis types saliva samples. The binding profile of VP8* proteins to HBGAs was identical for all the P[8] and P[4] strains tested, regardless of whether they were cell culture-adapted, vaccine or recent clinical strains (Fig. 2a). We observed a binding to mucins from secretor and Lewis positive (SE/Le^+^) individuals significantly greater than the one observed with mucins of individuals either secretor but Lewis negative (SE/le^−^), or non-secretor regardless of their Lewis phenotype (se/Le^+^ and se/le^−^) for which there was little or no binding at all (p < 0.001 or p < 0.01). Thus, the combination of SE and Le^+^ phenotypes appears to be the minimum requirement for P[8] and P[4] VP8* proteins to bind to human salivary mucins. SE/Le^+^ samples specifically contain the Lewis b difucosylated epitope (Fig. S1). Furthermore, direct binding of P[8] and P[4] VP8* to the difucosylated A Lewis b heptasaccharide was observed, whilst no binding occurred to related monofucosylated or non-fucosylated immobilized oligosaccharides, indicating requirement of the difucosylated Lewis b motif for binding (Fig. S2). Additionally, within the SE/Le^+^ group, the ABO phenotype showed an effect with a significantly greater binding to O blood group mucins compared to B blood group mucins (p < 0.01 or p < 0.05) (Fig. 2b). Most P[8] VP8* proteins (Rotarix, RotaTeq, Wa, 287 and 134) showed an enhanced recognition of A blood group mucins as compared to B blood group mucins (p < 0.001 or p < 0.05). Two P[8] VP8* proteins (287 and 183) revealed a stronger binding to O blood group mucins than to A blood group mucins (p < 0.01 and p < 0.05). Binding of the DS-1 P[4] strain to the A blood group antigen was previously reported^21^. Yet, we failed to reveal a higher binding to saliva from the A blood group secretor phenotype as compared to O or B phenotypes. Consistently, no binding to the monofucosylated A blood group antigen by the P[4] DS-1 was observed using neoglyconjugates (Fig. S2). Moreover, saliva samples from the A SE/le^−^ subtype were hardly recognized, unlike those from the A SE/Le^+^ subtype, indicating that the difucosylated motif, not the A epitope by itself, is the dominant binding epitope for the DS-1 strain, similar to the P[8] strains (Fig. S3). Collectively, these results indicate that expression of difucosylated structures, requiring both FUT2 and FUT3 enzymes for their expression, is essential for efficient attachment of P[8] and P[4] VP8* proteins. The effect of the ABO phenotype, with a binding to saliva samples from blood group O ≥ A > B, suggests hindrance of the difucosylated ligand by addition of the galactose and for some strains of the N-acetylgalactosamine residues that constitute the B and A antigens respectively.

### P[6] VP8* proteins bind to Lewis negative saliva samples

**Figure 3:**
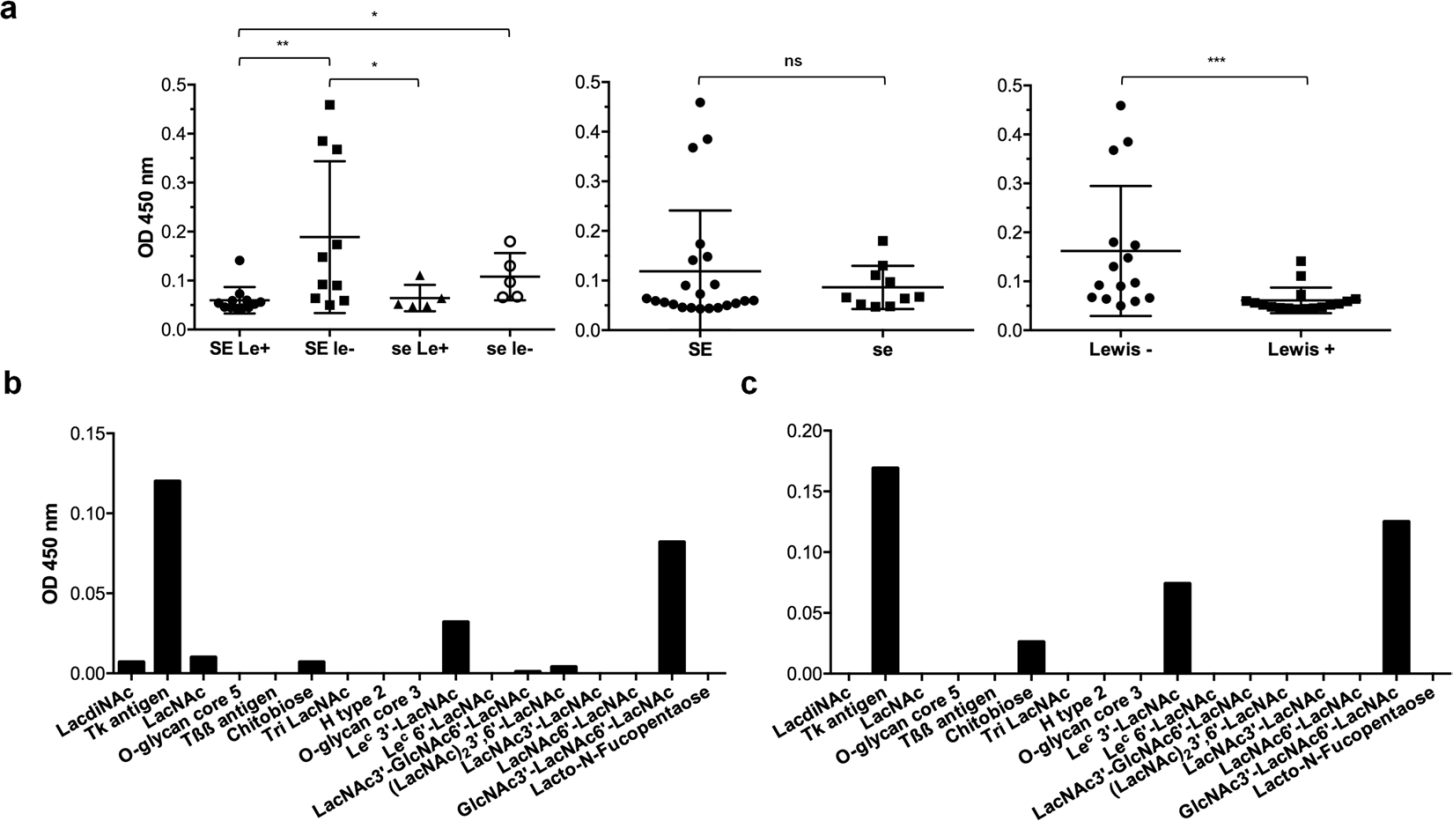
Binding of VP8* from P[6] strains isolated in Burkina Faso to HBGAs measured by saliva- and synthetic oligosaccharide-based binding assay. VP8* from a representative P[6] clinical strain (225) isolated in Burkina Faso was tested for binding to a panel of previously well-defined A, B, O, secretor and Lewis types saliva samples (SE = Secretor (FUT2^+^); se = non-secretor (FUT2^−^); Le^+^  = Lewis positive (FUT3^+^); le^−^ = Lewis negative (FUT3^−^)) (SE/Le^+^ n = 12, SE/le^−^ n = 10, se/Le^+^ n = 5 and se/le^−^ n = 5) (a) and a panel of synthetic oligosaccharides (b). To confirm the binding profile to oligosaccharides, VP8* from another P[6] isolate (268) was also tested (c). Mann-Whitney test was used to compare groups (***p < 0.001, **p < 0.01, *p < 0.05, ns = not significant).

**Figure 4:**
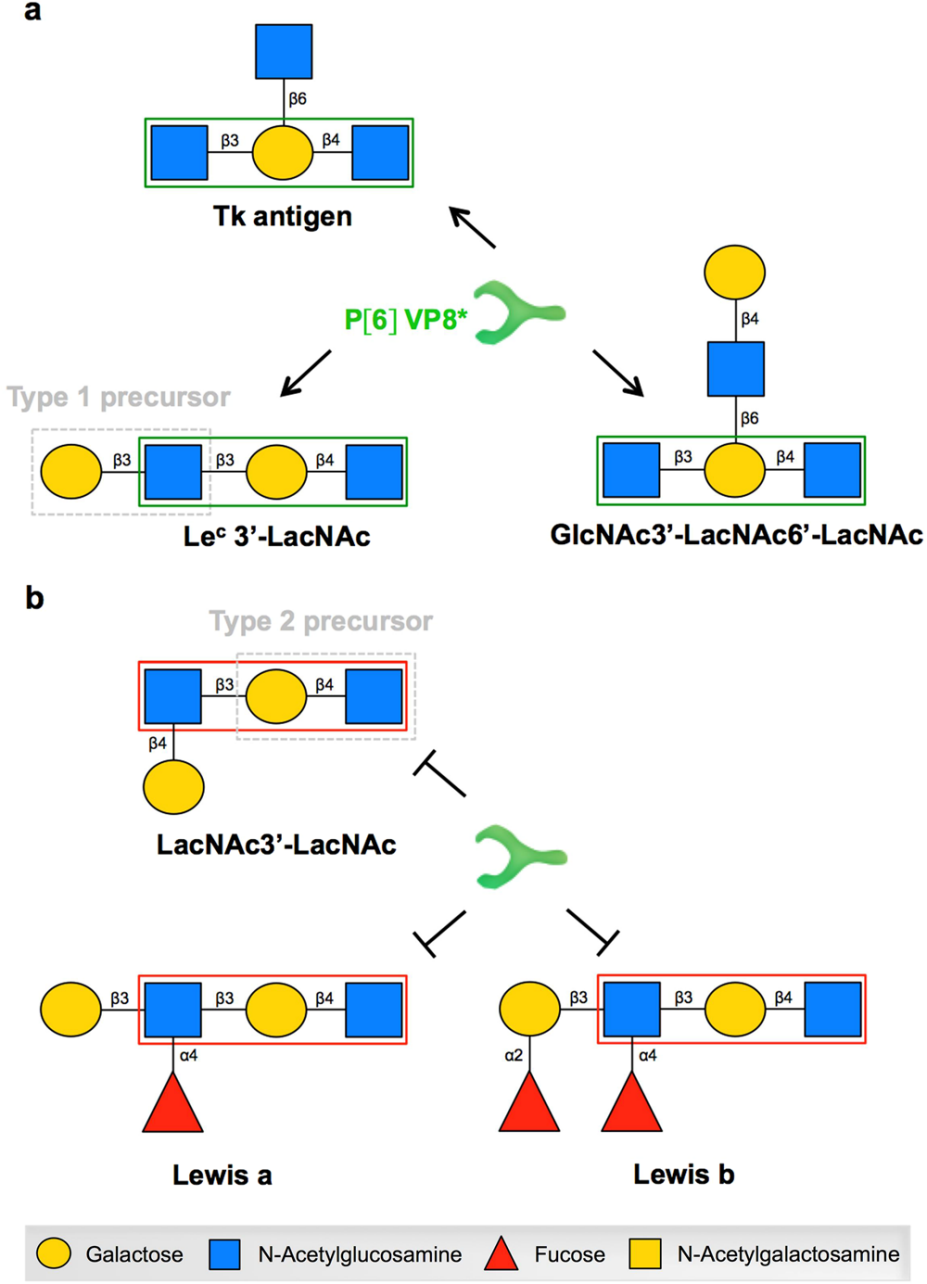
Proposed recognition pattern for P[6] VP8* proteins explaining the previously observed HBGA-dependant susceptibility to P[6] strains of RVs. **(a)** Schematic representation of the three oligosaccharides, including the HBGA type 1 precursor, recognized by P[6] VP8* proteins showing the proposed recognition pattern (green box). **(b)** Schematic representation of three oligosaccharides not recognized by P[6] VP8* proteins. These oligosaccharides have a substitution at position 4 of the subterminal N-acetylglucosamine. When the Lewis fucosyltransferase adds a fucose in α4 linkage to the galactose residue (making the Lewis a or Lewis b antigen), the VP8* recognition is lost.

Following P[8] and P[4], the P[6] genotype constitutes the third major P genotype of human RVs. Hence, we then investigated the binding of VP8* from P[6] clinical strains isolated in Burkina Faso to HBGAs by saliva- and synthetic oligosaccharide-based binding assays. We observed a preferential binding to Lewis negative saliva samples, irrespective of the secretor status (Fig. 3a). As this binding was not correlated with expression of the previously suggested H type 1 ligand (Fig. S4), we performed a synthetic oligosaccharide-based binding assay. This showed that P[6] VP8* proteins could bind to the Tk antigen, Le^c^ 3′-LacNAc and GlcNAc3′-LacNAc6′-LacNAc (Fig. 3b,c and Table S1). These synthetic sugars have in common a GlcNAcβ3Galβ4GlcNAc-R carbohydrate sequence that likely constitutes the main recognition pattern of these strains (Fig. 4). Substitution of its terminal N-acetylglucosamine at position 4, giving either a type 2 precursor or the Lewis antigen, was not tolerated. Yet, a substitution at position 3 that gives the type 1 precursor by addition of a galactose residue was well tolerated. Branching of the glycan chain generated by addition of an N-acetylglucosamine residue in β6 linkage did not appear to influence the binding either. Overall, these data strongly suggest that precursor structures, including the extended type 1 precursor Galβ3GlcNAcβ3Galβ4GlcNAc-R constitute the ligand of P[6] VP8* proteins. This motif is available in the highest amounts on mucins from Lewis negative individuals since it is not masked by addition of the α1,4-linked fucose by the FUT3 enzyme. Nonetheless, precursor structures remain available in all individuals due to incomplete glycan synthesis^38,39^. This likely explains why the difference in binding between Lewis positive and Lewis negative individuals was not absolute.

### P[14] VP8* proteins recognize mucins expressing the A antigen whereas P[2] VP8* proteins do not bind to human salivary mucins

**Figure 5:**
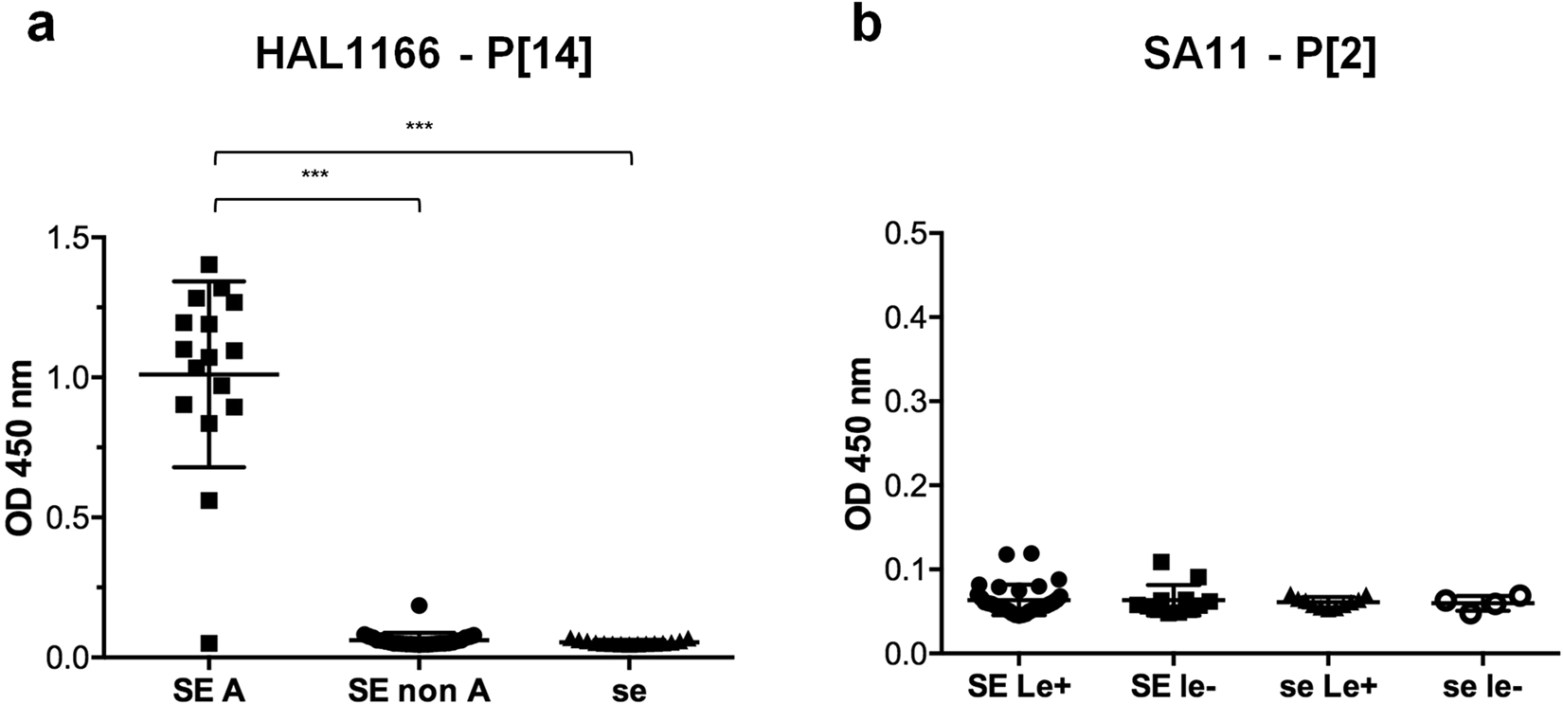
Binding of HAL1166 and SA11 VP8* to HBGAs measured by saliva-based binding assay. VP8* proteins from two cell culture-adapted strains (HAL1166 (P[14]) and SA11 (P[2])) were tested for binding to a panel of previously well-defined A, B, O, secretor and Lewis types saliva samples (SE = Secretor (FUT2 + ); se = non-secretor (FUT2^−^); Le^+^  = Lewis positive (FUT3^+^); le^−^ = Lewis negative (FUT3^−^); A = Blood group A). HAL1166 VP8* **(a)** binds to mucins of SE/A phenotypes only (SE non A n = 28, SE A n = 16 and se n = 15) whereas SA11 VP8* **(b)** does not bind to human salivary mucins (SE/Le^+^ n = 32, SE/le^−^ n = 12, se/Le^+^ n = 11 and se/le^−^ n = 4). Mann-Whitney test was used to compare groups (***p < 0.001).

We then extended the study to the binding of VP8* proteins from two cell culture-adapted strains: a P[14] sialidase-insensitive humain strain (HAL1166) and a P[2] sialidase-sensitive animal strain (SA11). HAL1166 VP8* proteins bound to saliva samples from blood group A secretor individuals only (Fig. 5a) and to the A blood group antigen on noeglycoconjugates (Fig. S2). By contrast, SA11 VP8* proteins did not recognize human mucins (Fig. 5b). To control that this absence of binding was not due to a loss of functionality, we tested the binding of SA11 VP8* to MA-104 cells by flow cytometry. As expected, SA11 VP8* binding was readily detected on MA-104 cells and abrogated by sialidase treatment (Fig. S5). Thus, SA11 VP8* failed to recognize human salivary mucins.

### Fucosylation is not a requirement for *in vitro* infection by P[8] and P[4] strains, unlike for infection of HT-29 cells by the P[14] HAL1166 strain

**Figure 6:**
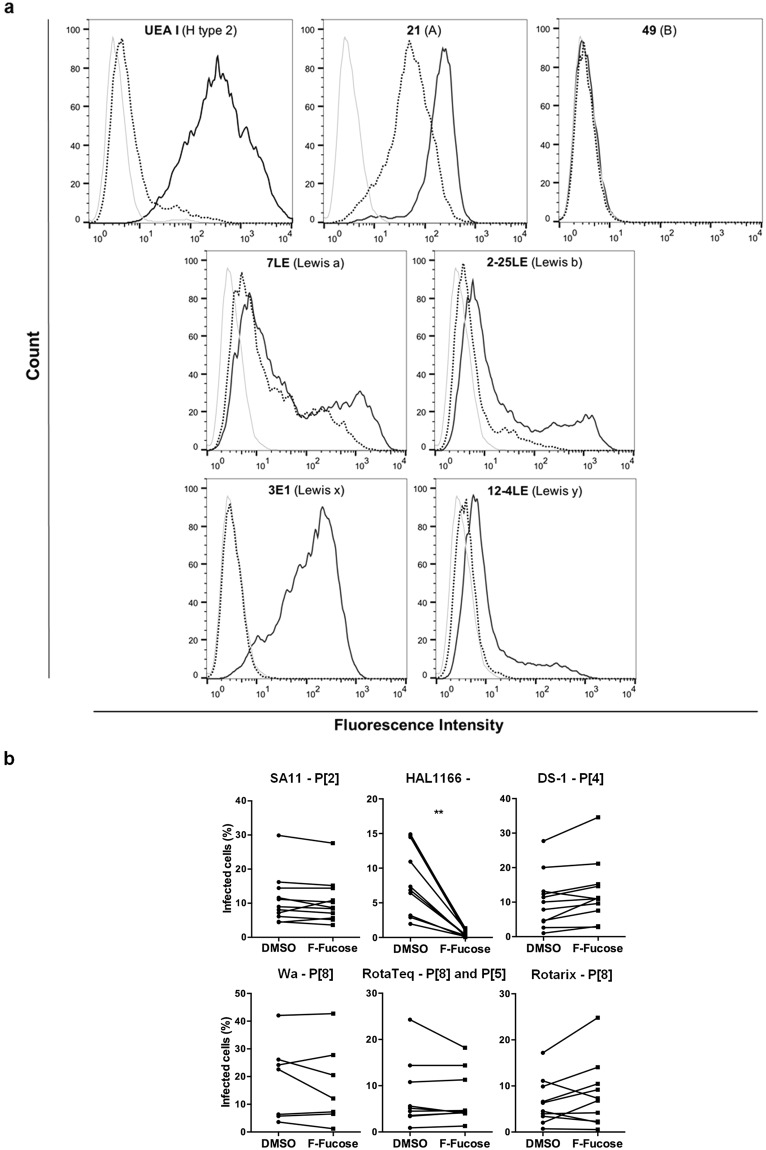
Effect of fucose synthesis blockade on *in vitro* infection of HT-29 cells. **(a)** 2F-fucose treatment efficacy was controlled by testing expression of various HBGAs (H type 2, A, B, Lewis a, Lewis b, Lewis x and Lewis y antigens) by flow cytometry: negative controls with secondary antibodies only (light grey); positive controls on DMSO treated cells (solid line); 2F-fucose treated cells (dotted line). The results provided are representative of those obtained from at least three independent experiments. **(b)** Infection of HT-29 cells, either DMSO (control) or 2F-fucose treated, by indicated cell culture-adapted strains of RV was quantified by fluorescence microscopy with an ArrayScan HCS Reader (Thermo Scientific). The results of each independent experiment are shown by linked control (DMSO) and treated (2F-fucose) values (SA11 n = 11; HAL1166 n = 10; DS-1 n = 11; Wa n = 7; RotaTeq n = 9 and Rotarix n = 10). Wilcoxon signed-rank test was used to compare the two groups (**p < 0.01).

**Figure 7:**
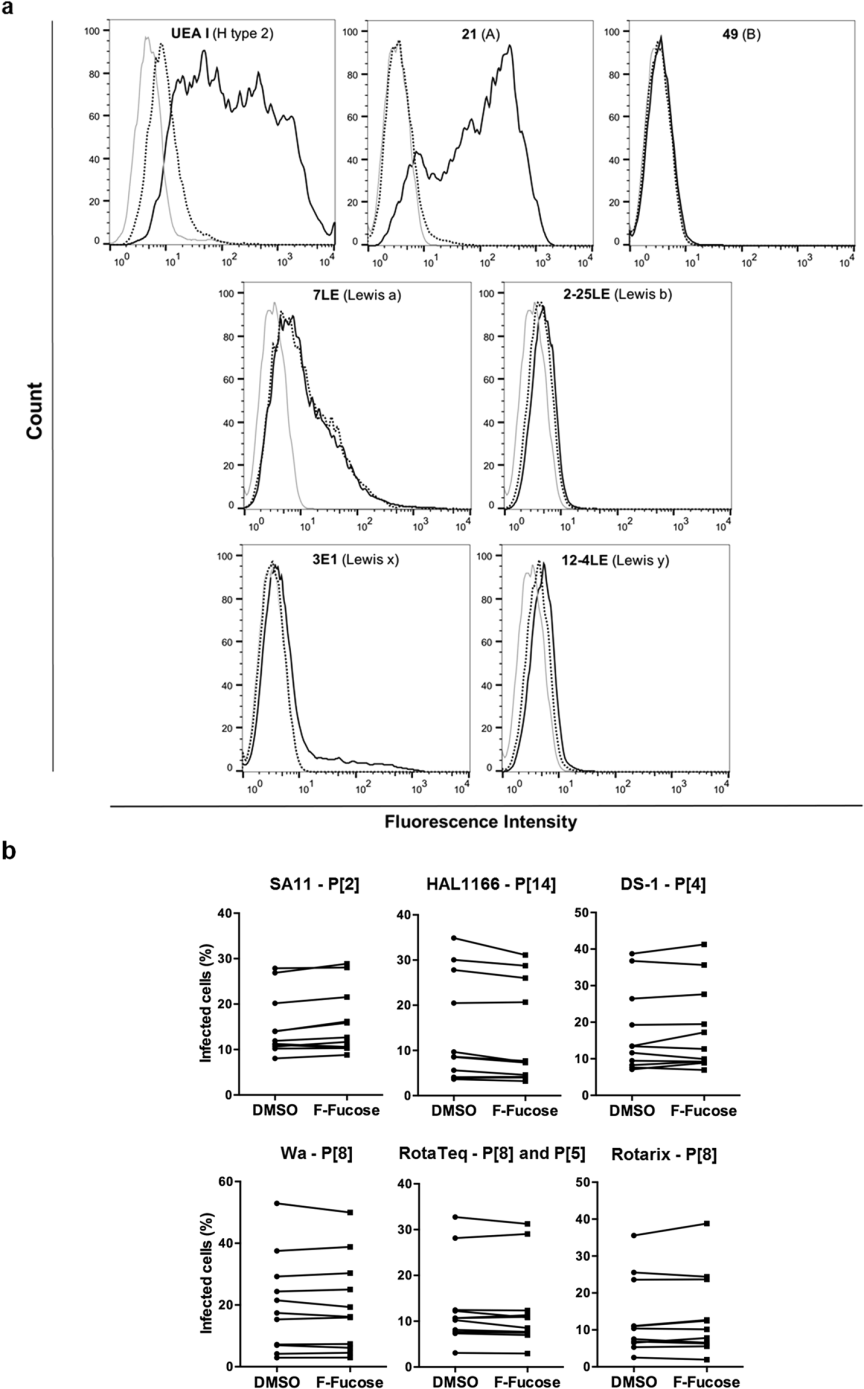
Effect of fucose synthesis blockade on *in vitro* infection of MA-104 cells. **(a)** 2F-fucose treatment efficacy was controlled by testing expression of various HBGAs (H type 2, A, B, Lewis a, Lewis b, Lewis x and Lewis y antigens) by flow cytometry: negative controls with secondary antibodies only (light grey); positive controls on DMSO treated cells (solid line); 2F-fucose treated cells (dotted line). The results provided are representative of those obtained from at least three independent experiments. **(b)** Infection of MA-104 cells, either DMSO (control) or 2F-fucose treated, by indicated cell culture-adapted strains of RV was quantified by fluorescence microscopy with an ArrayScan HCS Reader (Thermo Scientific). The results of each independent experiment are shown by linked control (DMSO) and treated (2F-fucose) values (n = 11 for each strain of RV).

In order to gain a better understanding of the contribution of HBGAs in the infection process, we then used an *in vitro* infection model. First, the expression of HBGAs on susceptible cells (HT-29, MA-104, Vero and Caco-2) was blocked using 2F-fucose, a metabolic inhibitor of fucosylation. Treatment efficacy was controlled by assessing expression of various HBGAs by flow cytometry. Control HT-29 cells, cultured in the presence of DMSO, express H, A and Lewis antigens. Expression of these structures on 2F-fucose-treated HT-29 cells was fully or substantially suppressed (Fig. 6a). Similar experiments conducted on MA-104, Vero and Caco-2 cells allowed us to draw the same conclusion, although initial phenotypes were different (Figs 7a, S6 and S7). Infection of these cells, either DMSO- (control) or 2F-fucose-treated, by several cell culture-adapted strains of RVs was then quantified by fluorescence microscopy. Infection by the sialic acid-dependent SA11 animal strain of P[2] genotype was used as control. It confirmed that fucosylation was not needed for infection by this strain since no significant decrease in infection was detected (Figs 6b, 7b and S6, S7). For the P[14] HAL1166 strain, there was a significant decrease in infection of 2F-fucose-treated HT-29 cells compared to control cells. Yet, that was not the case for the other strains (DS-1 P[4], Wa P[8], Rotarix P[8] and RotaTeq P[8] and P[5]) (Fig. 6b). Finally, no matter what strain was used, 2F-fucose treatment of other susceptible cell lines (MA-104, Vero and Caco-2) had no effect on infection (Figs 7b, S6 and S7). These data indicate that fucosylation and thereby expression of HBGAs is not a requirement for *in vitro* infection by P[8] and P[4] strains on transformed cells of non-small intestinal origin. However, in accordance with a previous study showing abrogation of virus infectivity in HT-29 cells by anti-A type antibodies^22^, our data indicate that fucosylation and more specifically synthesis of the A antigen is essential for *in vitro* infection by the P[14] HAL1166 strain. In agreement with the latter result, infection by the P[14] HAL1166 strain of Vero and Caco-2 cells that do not express the A antigen was very low. Intriguingly, infection by HAL1166 of MA-104 cells expressing the A antigen, like HT-29 cells, was not altered by its complete loss upon 2F-fucose treatment. Thus, the A antigen can be considered as virus receptor for P[14] strains on HT-29 cells, but not on MA-104 cells.

### The presence of neutralizing antibodies is related to the presence of the wild type *FUT2* allele

**Figure 8:**
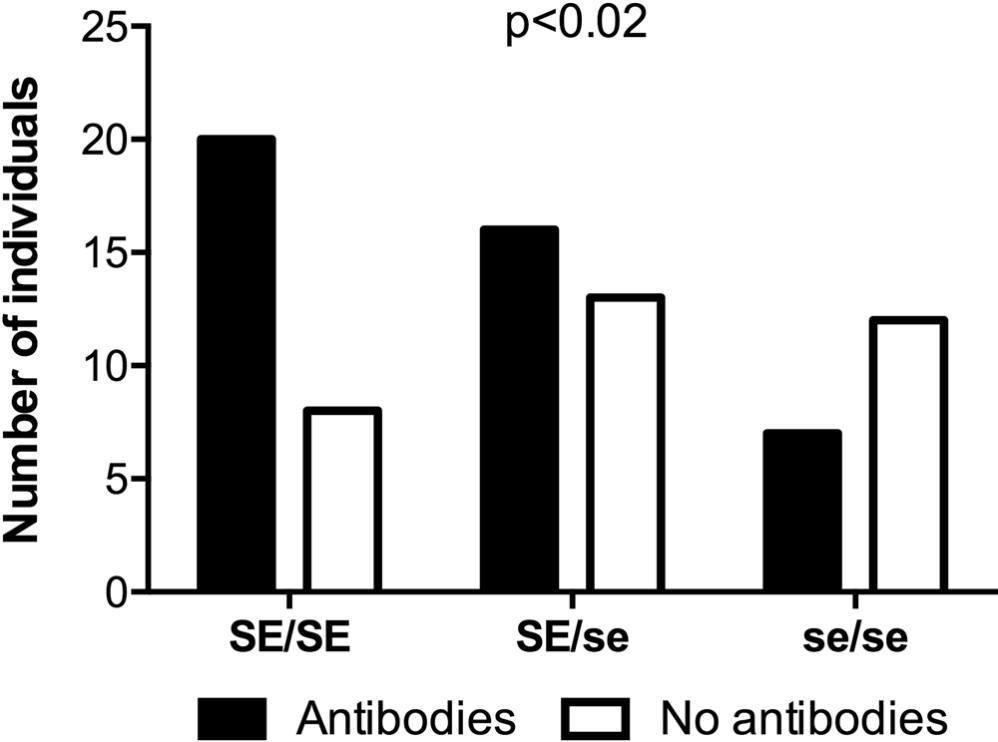
Relationship between the presence of neutralizing antibodies to the human RV strain Wa (P[8]) and the *FUT2* genotype in French healthy young adults. Presence of neutralizing antibodies against the RV strain Wa (P[8]) was determined in sera from 76 healthy individuals by serum virus neutralization assay. The distribution of each genotype was as follows: 28 homozygous secretors (SE/SE); 29 heterozygous secretors (SE/se) and 19 non-secretors (se/se). Titers were defined as the dilution of serum leading to a 50% inhibition of infection as compared to the positive control. Two groups of individuals were then defined: those for whom a titer was obtained and those for whom no titer was obtained. Chi-square test for trend was used to compare distributions (p < 0.02).

Finally, we investigated the role of *FUT2* genotypes in the neutralizing antibody response against the Wa human strain of P[8] genotype. Presence of neutralizing antibodies was determined by serum virus neutralization assay for 76 young healthy adults. Serum neutralizing antibodies as defined by a 50% inhibition above a 1/20 serum dilution, were present in 43 samples (57%), whereas no inhibition or inhibition below the threshold was observed for 33 samples (43%). The distribution of these two groups (with or without neutralizing antibodies) within the 3 *FUT2* genotypes was then assessed. The number of individuals displaying neutralizing antibodies was significantly associated with the presence of the *FUT2* wild type allele (p < 0.02) (Fig. 8). This observation indicates that the *FUT2* genetic polymorphism plays a role in the susceptibility to RV infection that gives a long-lasting *in vitro* neutralizing antibody response.

## Discussion

A series of recent epidemiological studies conducted in France^24^, Vietnam^25^, Burkina Faso^26^, USA^27^ and China^20^ showed that only children of the secretor geno- or phenotype, thus having at least one functional *FUT2* allele, were found among those suffering from gastroenteritis caused by P[8] and P[4] RVs. Additionally, the study conducted in Burkina Faso, showed that no Secretor/Lewis negative child with gastroenteritis was infected by P[8] strains^26^. Moreover, the same study indicated that in case of infection by P[6] strains, Lewis negative children were at a greater risk of being among patients than Lewis positive children, regardless of their secretor status. These concordant data provide strong evidence that *FUT2* (secretor) and *FUT3* (Lewis) genetic polymorphisms contribute to susceptibility to major strains of RVs and therefore that HBGAs are involved in the infection process. Nonetheless, how and where in the body this takes place remains unclear.

In the present study, we produced VP8* proteins from clinical, vaccine and cell culture-adapted strains of several P genotypes, looking for relationships between their epidemiological profiles and their binding patterns to saliva samples of different HBGA types. There was no difference between the HBGA binding profiles of clinical and cell culture-adapted P[8] strains, indicating that HBGA binding was not a cell culture adaptation. We documented that all P[8] and P[4] VP8* proteins require both the secretor and Lewis fucosyltransferases (FUT2 and FUT3) for binding to saliva, indicating recognition of difucosylated structures. This is consistent with earlier results showing binding of P[8] and P[4] VP8* proteins to the Lewis b antigen^17^ and to saliva samples from secretor individuals^18–20^. Interestingly, Huang *et al*.^17^ observed a binding to the H type 1 antigen for P[8] and P[4] strains. However, saliva samples of secretor Lewis negative individuals (expressing the H type 1 antigen, but not the Lewis b antigen) were not recognized in our study. It is important to note that in the same study Huang *et al*.^17^ observed specific binding of purified Wa triple layer particles to secretor Lewis b positive, but not to secretor Lewis b negative saliva samples, suggesting that binding to synthetic oligosaccharides may not perfectly mimic the natural ligands found on mucins. Regardless, the match between the results from the saliva binding assay and the epidemiological data suggests that the difucosylated Lewis b antigen may be a functional ligand *in vivo* for P[8] strains. P[8] and P[4] strains are the most commonly encountered worldwide and among the HBGA subgroups, the secretor and Lewis positive subgroup, which is preferentially recognized by P[8] and P[4] VP8*, is the most common (around 72–76% of the population in Europe). Behind P[8] and P[4], the P[6] genotype constitutes the third major P genotype of human RVs. Hence, we then investigated the binding of VP8* from two P[6] strains isolated in Burkina Faso. We observed that P[6] VP8* proteins bind preferentially to Lewis negative saliva samples, irrespective of the secretor status. This is consistent with findings of the epidemiological study conducted in Burkina Faso which showed that these same P[6] strains caused disease among Lewis negative children mainly, independent of their secretor status^26^, again illustrating the match between the saliva binding assay and epidemiological data. We observed that the glycan motif recognized by these clinical P[6] strains is a precursor structure comprised in the type 1 precursor Galβ3GlcNAcβ3Galβ4GlcNAc, although the terminal galactose does not appear to contribute much to the binding. Conversion into Lewis antigens by the FUT3 enzyme, as occurs in Lewis positive individuals, impaired recognition, explaining the lower binding to saliva of this phenotype. Our results are also compatible with those of previous studies showing binding to a group of long glycans containing the type 1 precursor HBGA terminal sequences, with or without the H epitope, but without the Lewis epitope^40,41^. Another previous study reported binding of the VP8* from a P[6] strain to A blood group oligosaccharide in solution^21^ which contradicts the epidemiological data where a lower frequency of the A blood group was found among ill children infected by P[6] strains in comparison with the O blood group^26^. This discrepancy may be due to the use of an oligosaccharide in solution or of a cell culture-adapted strain. P[6] strains that have been most frequently found in individuals of the Lewis negative phenotype are seldom encountered in Europe where this phenotype represents only 5–10% of the population. Interestingly, its frequency can be as high as 30% in some Latino-American and African populations^42,43^ where those strains are more common. Since mucins from Lewis positive individuals also contain the un-substituted type 1 precursor, albeit in lower amount than Lewis negative individuals^44^, Lewis positive children may also be infected, although less frequently, increasing to some extent the pool of susceptible individuals in the population.

We then extended the study to the binding of VP8* proteins from two cell culture-adapted strains: the HAL1166 sialidase-insensitive human strain of P[14] genotype and the SA11 sialidase-sensitive animal strain of P[2] genotype. We found that the HAL1166 VP8* proteins exclusively recognize mucins expressing the A antigen. This is in agreement with other studies showing that P[14] VP8* proteins recognize the type A HBGA^22,23^. As previously suggested by Liu *et al*.^26^, the fact that P[14] strains recognize the A antigen, which is a common carbohydrate shared between species, could explain why they are capable of causing disease to both human and other mammals. P[9], P[14] and P[25] strains exclusively recognize A blood group individuals who represent between 8–35% of the population depending on geographical areas^45^. Although, as yet, there is no epidemiological evidence indicating that only A blood group secretors are susceptible to disease due to these strains, it is likely that the limited frequency of this phenotype is too low to sustain transmission in humans, therefore explaining why they are seldom encountered. Collectively these observations point to a relationship between the frequency of genetically susceptible individuals in the population and the circulation of the viral strains.

VP8* from the SA11 sialidase-sensitive animal strain did not bind to human salivary mucins. N-acetylneuraminic acid (Neu5Ac) and N-glycolylneuraminic acid (Neu5Gc) are the most common sialic acid family members with Neu5Gc differing from Neu5Ac by an additional hydroxyl group^46^. Interestingly, a previous study showed that Triple-Layered Particles (TLPs) from the SA11 strain strongly bind to the ganglioside NeuGc-GM3, while they do not bind to NeuAc-GM3^47^. Therefore, the lack of binding to human saliva could be due to the absence of NeuGc, which is not expressed in normal human tissues but can be found in most mammalian tissues^48^. This could explain why P[2] strains can infect a wide variety of animal species, whilst the lack of binding to human saliva is associated with the lack of human infection^26^.

In addition to the glycan specificity of P[8] and P[4] strains VP8*, we also investigated the relationship between the RV-specific neutralizing antibodies in sera from healthy French blood donors and their *FUT2* genotype. The presence of neutralizing antibodies is the result of previous infections and constitutes an indirect marker of virus susceptibility. Here, we showed that the probability of having neutralizing antibodies was associated with the presence of the *FUT2* wild type allele. This is concordant with results of previous studies^20,29,30^ suggesting that individuals with a secretor phenotype might be more susceptible to disease caused by RV infections as compared to non-secretors. Nonetheless, the fact that non-secretor individuals also exhibited neutralizing antibodies suggests that children with this HBGA phenotype might be somewhat susceptible to infection by P[4] or P[8] strains that constitute the vast majority of strains circulating in France^49^, or they might have been infected by strains of other P types that generated heterotypic antibodies. An alternative explanation for the observed relationship between the *FUT2* polymorphism and the RV-specific neutralizing antibodies levels might stem from the impact of the *FUT2* polymorphism on the microbiota composition that in turn would influence the antibody response to RV infection^50^. Several reports described associations between the *FUT2* genotype and the gut microbiota composition^30,51–53^. Yet, recent studies performed on large cohorts failed to confirm these associations^54,55^. Nonetheless, the microbiota composition of our cohort of 75 volunteers was determined. No significant association was found with the *FUT2* genotypes, ruling out this possibility (to be published elsewhere).

The fact that the HBGA recognition is conserved between recent (clinical strains) and older (Wa and vaccine strains) P[8] strains suggests that HBGAs polymorphism may contribute to explain the modest efficacy of vaccines in areas where the frequency of FUT2 negative (non-secretor) and/or FUT3 negative (Lewis negative) individuals is high since these are areas where strains of other P types such as P[6] circulate most readily, as discussed above. It is also interesting to note that within the secretor/Lewis positive subgroup, we observed an effect of the ABO phenotype on binding to saliva samples of all P[8] strains. These results are in agreement with a previous study showing a lower binding of P[8] VP8* to blood type B saliva samples, suggesting also hindrance by addition of the galactose residue constituting the B antigen^17^. Interestingly, two recent studies conducted in Pakistan and Nicaragua, reported lower seroconversion rates after vaccination in nonsecretors as well as in non-blood group O secretors or B blood group secretors respectively^56,57^. The concordance of these new observations with the HBGA specificity of the vaccines VP8* strongly suggests that the *FUT2* and *ABO* gene polymorphisms might impact the quality of immunization. Further epidemiological studies would be necessary to confirm these results and clarify whether the combinations of *ABO*, *FUT2* and *FUT3* subtypes and their variable frequencies among populations can contribute to explain the lower efficacy of RVA vaccines in some countries.

Since as described above, multiple studies point towards an essential role of HBGA-binding in the infection process, we studied the effect of fucose synthesis blockade on *in vitro* infection. That led us to conclude that fucosylation is essential for *in vitro* infection of HT-29 cells by the P[14] HAL1166 strain but it is not a requirement for infection of various cell lines by P[8] and P[4] strains. These findings suggest that HBGA-binding could be an early event which is not involved in the *in vitro* infection of poorly differentiated cells. Alternatively, the presence of HBGAs might enhance viral replication and development of the disease but not be essential for infection in the strict sense. The use of more sophisticated cellular models such as Human Intestinal Enteroids (HIEs), will be necessary to clarify this point^58,59^.

In conclusion, our findings further indicate that human HBGAs expressed on host cells or digestive tissues may be used by the major P genotypes of circulating RVs as attachment factors or receptors. Alongside epidemiological data, this suggests that genetically controlled polymorphic human HBGAs affect the phylogeographic distribution of these viruses. Differential expression or frequency of HBGAs could also be of major importance in the host tropism and transmission of RVs between human and animal species. More experimental data are required to elucidate the mechanisms whereby HBGAs impact RV infection and disease. These data would be of primary interest for optimization of current vaccines or development of new vaccine strategies.

## Methods

### Production of VP8* proteins

Plasmids expressing the gene encoding the VP8* core proteins were synthesized by GeneART (Rotarix (P[8]), RotaTeq (P[8] strain) and SA11 (P[2])), cloned from cell culture-adapted virus (HAL1166 (P[14]), DS-1 (P[4]) and Wa (P[8])) or directly isolated from clinical stool samples (French isolates 134, 183, 287 (P[8]) and Burkinabe isolates 225 and 268 (P[6])) (see supplementary methods for GenBank accession numbers) as previously described^17^. In brief, complementary DNA produced from viral dsRNA by reverse transcription was used as template for PCR with specific primers to produce the VP8* gene fragment. VP8* gene fragments were cloned into the expression vector pGEX-4T-1 (GE Healthcare Life Sciences) between the SalI and BamHI sites. The final expression plasmid encoded for amino acids 46 to 224 of the VP4 protein fused to an N-terminal GST protein. After confirmation of the inserts through DNA sequencing, protein expression was performed mostly as described previously^17^. Expression of recombinant proteins by *Escherichia coli* strain BL21 (DE3) (Novagen) was induced at 22 °C overnight by isopropyl β-D-thiogalactopyranoside at a final concentration of 0.4 mM. Frozen cell pellets were thawed in PBS. GST fusion proteins were sequentially extracted/purified from cells as follows: sonication/chemical lysis (1% Triton X-100), centrifugation, GSTrap column capture and ion exchange chromatography purification. The possible high-molecular weight (MW) complex formation was examined using size exclusion gel filtration (Superdex 200, GE Healthcare Life Sciences). Fractions of intermediate size were used for further work and kept at −20 °C. The concentration of the purified VP8* was determined by measuring absorbance at 280 nm.

### VP8* binding to HBGAs

The binding of VP8* to HBGAs was measured by saliva- and synthetic oligosaccharide-based binding assays. A panel of previously well-defined A, B, O, secretor and Lewis types saliva samples was used as source of HBGAs (see supplementary methods for more information) in the saliva-based binding assays which were performed mostly as described previously^23^. Briefly, boiled saliva samples were diluted at 1:1,000 in 0.1 M carbonate-bicarbonate buffer pH 9.6 and coated onto 96-well microtiter plates (NUNC MaxiSorp) at 4 °C overnight. For the oligosaccharide-based binding assays, polyacrylamide neoglycoconjugates (IBCh, Russia), which are listed on Table S1, were coated at 20 μg/ml. After blocking with PBS containing 5% nonfat milk, GST-VP8* protein at 5 to 20 μg/ml was added and incubated at 4 °C overnight. The bound VP8* proteins were detected using a rat anti-serum to VP8* (see supplementary methods and Fig. S8 for more information) at 1:500 followed by HRP-conjugated goat anti-rat IgG (Sigma-Aldrich) at 1:5,000. In each step, the plates were incubated for 1 h at 37 °C and washed three times with PBS − 0.05% Tween 20. The signal intensities were displayed using a 3,3′,5,5′-Tetramethylbenzidine kit (BD OptEIA, BD Biosciences) and the absorbance was measured at 450 nm using a microplate absorbance reader (iMark, Bio-Rad). For the saliva-based binding assays, raw absorbance data are shown and background noise was comprised between 0.05 and 0.1. For the synthetic oligosaccharide-based binding assays, background noise was subtracted from raw data and was around 0.1. Saliva samples collection was approved by the Nantes University Hospital Review Board (study no. BRD02/2-P), informed consent was obtained from all saliva donors and all experiments were performed in accordance with the French national guidelines and regulations.

### Infectivity assay

A description of cells and viruses used in this study is provided in supplementary methods. RV infectivity was activated with porcine pancreatic trypsin (Sigma) at 10 µg/ml for 1 h at 37 °C. Cells were washed twice with serum-free medium and inoculated with trypsin-activated virus at a multiplicity of infection (MOI) ranging between 0.5 and 5 depending on the cell type. Plates were then incubated at 37 °C for 45–90 min. After allowing virus attachment to cells, the inoculum was removed and serum-supplemented medium was added. The infection was left to proceed for 14–15 h at 37 °C in 95% (vol/vol) air with 5% (vol/vol) CO_2_. Infected cells in methanol-fixed cell monolayers were detected by staining cells using a goat polyclonal anti-RV serum (Bio-Rad Antibodies) and FITC-labeled rabbit anti-goat IgG (Fc) antibody (Bio-Rad Antibodies), both diluted at 1:400 in PBS containing 3% BSA. Cell nuclei were stained with DAPI. Plates reading was performed on an ArrayScan HCS (ThermoScientific) which gives percentages of infected cells after counting the total cell number (number of blue elements) and the infected-cell number (number of both blue and green elements).

### Treatment of cells with 2F-fucose

To inhibit the synthesis of fucosylated structures, a competitive inhibitor of fucosyltransferases, the 2F-Peracetyl-Fucose (Merck) was used. This inhibitor enters the cell by passive diffusion and is metabolized into a GDP-fucose analog (2F-fucose) to form an inhibitory substrate of fucosyltransferases^60^. Cells were seeded in 96-well plates or 25 cm² flasks and treated with 500 µM 2F-Peracetyl-Fucose (Merck) solubilized in DMSO in the adequate medium for 3 days, replacing the medium with fresh drug daily. Negative controls were performed by adding the DMSO vehicle only to the culture medium. Afterwards, treated cells were phenotyped by flow cytometry or infected by RVs.

### Flow cytometry phenotyping

MA-104, HT-29, Caco-2 and Vero cells from confluent cell monolayers were detached by brief treatment with trypsin-EDTA and resuspended in the appropriate culture medium containing 10% (vol/vol) fetal bovine serum. The following reactions were performed with viable cells (2.10^5^ cells/well of a 96-well plate) at 4 °C and incubation time was 30 min. The presence of histo-blood group H, A, B, Lewis a, Lewis b, Lewis x and Lewis y antigens at the cell surface was detected by indirect immunofluorescence staining (see supplementary methods for more information). Cell binding of GST-VP8* from SA11 was assayed as followed. GST-VP8* at 40 µg/ml was reacted with cells for 1 h. Cell-bound protein was detected with a rat anti-serum to VP8* diluted at 1:1,000. Similarly diluted normal rat serum served as a negative control. Bound rat antibodies were detected with FITC-conjugated goat anti-rat IgG (H + L) (Thermo Fisher Scientific) diluted at 1:200. After final washes in PBS containing 0.1% BSA and PBS only, flow-cytometry acquisition was performed on a BD Accuri C6 flow cytometer (BD Biosciences) and data were analysed using FlowJo software. The level of cell surface expression or GST-VP8* bound to cells was expressed as the mean fluorescence intensity (MFI).

### Serum samples

Serum samples used in this study come from the GOMMS PRL12009 project as a part of the biobank of Biofortis MérieuxNutriSciences. This biocollection is registered at the French Research Ministry (AC-2013–1792) and the PRL12009 project was approved by the French Ethic Comity (CPP Ouest IV). All volunteers were aware of the protocol of the study and fulfilled the informed consent form. They were aged 18–30 years and considered as healthy.

### Serum virus neutralization assay

Sera from individuals of known ABO, secretor, and Lewis types (typed as previously described^24,61^) were initially diluted at 1:20 using serum-free medium. Then, two-fold dilutions of all samples were made to reach a final dilution of 1:320. Simultaneously, infectivity of the human Wa strain was activated as described above. Serially diluted sera were pre-incubated with 2 × 10^3^ FFU of the virus for 1.5 h at 37 °C before addition to MA-104 cells.

### Statistical analysis

GraphPad Prism v 5.0 (GraphPad Software, San Diego, CA, USA) was used for data analysis. Comparisons for evaluation of VP8* proteins binding, cell infection and neutralization antibodies were performed by Mann-Whitney test, Wilcoxon signed-rank test, based on median values and Chi square test for trend, respectively. Differences were considered statistically significant when the level of two-tailed significance was p < 0.05.

## Electronic supplementary material

Supplementary Information

## Data Availability

All data analyzed during this study are included in this published article (and its Supplementary Information files).

## References

[1] Pesavento JB, Crawford SE, Estes MK, Prasad BVV (2006). Rotavirus proteins: structure and assembly. Curr. Top. Microbiol. Immunol..

[2] Matthijnssens J (2011). Uniformity of rotavirus strain nomenclature proposed by the Rotavirus Classification Working Group (RCWG). Arch. Virol..

[3] Santos N, Hoshino Y (2005). Global distribution of rotavirus serotypes/genotypes and its implication for the development and implementation of an effective rotavirus vaccine. Rev. Med. Virol..

[4] Desselberger U (2014). Rotaviruses. Virus Res..

[5] Bányai K (2012). Systematic review of regional and temporal trends in global rotavirus strain diversity in the pre rotavirus vaccine era: insights for understanding the impact of rotavirus vaccination programs. Vaccine.

[6] Settembre EC, Chen JZ, Dormitzer PR, Grigorieff N, Harrison SC (2011). Atomic model of an infectious rotavirus particle. EMBO J..

[7] Padilla-Noriega L, Dunn SJ, López S, Greenberg HB, Arias CF (1995). Identification of two independent neutralization domains on the VP4 trypsin cleavage products VP5* and VP8* of human rotavirus ST3. Virology.

[8] Lopez S, Arias CF (2006). Early steps in rotavirus cell entry. Curr. Top. Microbiol. Immunol..

[9] Zárate S (2000). The VP5 Domain of VP4 Can Mediate Attachment of Rotaviruses to Cells. J. Virol..

[10] Wolf M, Vo PT, Greenberg HB (2011). Rhesus rotavirus entry into a polarized epithelium is endocytosis dependent and involves sequential VP4 conformational changes. J. Virol..

[11] Ciarlet M, Estes MK (1999). Human and most animal rotavirus strains do not require the presence of sialic acid on the cell surface for efficient infectivity. J. Gen. Virol..

[12] Haselhorst T (2009). Sialic acid dependence in rotavirus host cell invasion. Nat. Chem. Biol..

[13] Fleming FE (2014). Relative roles of GM1 ganglioside, N-acylneuraminic acids, and α2β1 integrin in mediating rotavirus infection. J. Virol..

[14] Arias, C. F., Silva-Ayala, D., Isa, P., Díaz-Salinas, M. A. & López, S. Chapter 2. 2 - Rotavirus Attachment, Internalization, and VesicularTraffic. In *ViralGastroenteritis* (eds Svensson, L., Desselberger, U., Greenberg, H. B. & Estes, M. K.) 103–119 (Academic Press, 10.1016/B978-0-12-802241-2.00006-7 (2016).

[15] Clausen H, Hakomori S (1989). ABH and related histo-blood group antigens; immunochemical differences in carrier isotypes and their distribution. Vox Sang..

[16] Marionneau S (2001). ABH and Lewis histo-blood group antigens, a model for the meaning of oligosaccharide diversity in the face of a changing world. Biochimie.

[17] Huang P (2012). Spike Protein VP8* of Human Rotavirus Recognizes Histo-Blood Group Antigens in a Type-Specific Manner. J. Virol..

[18] Ma X (2015). Binding Patterns of Rotavirus Genotypes P[4], P[6], and P[8] in China with Histo-Blood Group Antigens. PloS One.

[19] Sun X (2016). Binding specificity of P[8] VP8* proteins of rotavirus vaccine strains with histo-blood group antigens. Virology.

[20] Zhang X-F (2016). P[8] and P[4] Rotavirus Infection Associated with Secretor Phenotypes Among Children in South China. Sci. Rep..

[21] Böhm R (2015). Revisiting the role of histo-blood group antigens in rotavirus host-cell invasion. Nat. Commun..

[22] Hu L (2012). Cell attachment protein VP8* of a human rotavirus specifically interacts with A-type histo-blood group antigen. Nature.

[23] Liu Y (2012). Rotavirus VP8*: Phylogeny, Host Range, and Interaction with Histo-Blood Group Antigens. J. Virol..

[24] Imbert-Marcille B-M (2014). A FUT2 gene common polymorphism determines resistance to rotavirus A of the P[8] genotype. J. Infect. Dis..

[25] Van Trang N (2014). Association between norovirus and rotavirus infection and histo-blood group antigen types in Vietnamese children. J. Clin. Microbiol..

[26] Nordgren J (2014). Both Lewis and secretor status mediate susceptibility to rotavirus infections in a rotavirus genotype-dependent manner. Clin. Infect. Dis. Off. Publ. Infect. Dis. Soc. Am..

[27] Payne DC (2015). Epidemiologic Association Between FUT2 Secretor Status and Severe Rotavirus Gastroenteritis in Children in the United States. JAMA Pediatr..

[28] Yang T-A, Hou J-Y, Huang Y-C, Chen C-J (2017). Genetic Susceptibility to Rotavirus Gastroenteritis and Vaccine Effectiveness in Taiwanese Children. Sci. Rep..

[29] Günaydın G, Nordgren J, Sharma S, Hammarström L (2016). Association of elevated rotavirus-specific antibody titers with HBGA secretor status in Swedish individuals: The FUT2 gene as a putative susceptibility determinant for infection. Virus Res..

[30] Rodríguez-Díaz J (2017). Relevance of secretor status genotype and microbiota composition in susceptibility to rotavirus and norovirus infections in humans. Sci. Rep..

[31] Ayouni S (2015). Rotavirus P[8] Infections in Persons with Secretor and Nonsecretor Phenotypes, Tunisia. Emerg. Infect. Dis..

[32] Tate JE (2012). 2008 estimate of worldwide rotavirus-associated mortality in children younger than 5 years before the introduction of universal rotavirus vaccination programmes: a systematic review and meta-analysis. Lancet Infect. Dis..

[33] Walker CLF (2013). Global burden of childhood pneumonia and diarrhoea. The Lancet.

[34] Angel J, Franco MA, Greenberg HB (2007). Rotavirus vaccines: recent developments and future considerations. Nat. Rev. Microbiol..

[35] Glass RI, Parashar U, Patel M, Gentsch J, Jiang B (2014). Rotavirus vaccines: successes and challenges. J. Infect..

[36] López CA, Sovova Z, van Eerden FJ, de Vries AH, Marrink SJ (2013). Martini Force Field Parameters for Glycolipids. J. Chem. Theory Comput..

[37] Ruvoën-Clouet N (2014). Increase in genogroup II.4 norovirus host spectrum by CagA-positive Helicobacter pylori infection. J. Infect. Dis..

[38] Thomsson KA (2002). The salivary mucin MG1 (MUC5B) carries a repertoire of unique oligosaccharides that is large and diverse. Glycobiology.

[39] Mihalache, A. *et al*. Structural Characterization of Mucin O-Glycosylation May Provide Important Information to Help Prevent Colorectal Tumor Recurrence. *Front*. *Oncol*. **5** (2015). 10.3389/fonc.2015.00217 PMC4597131 26500890

[40] Yu Y (2014). Human milk contains novel glycans that are potential decoy receptors for neonatal rotaviruses. Mol. Cell. Proteomics MCP.

[41] Liu Y (2016). Glycan Specificity of P[19] Rotavirus and Comparison with Those of Related P Genotypes. J. Virol..

[42] Corvelo TCO, Aguiar DCF, Sagica FES (2002). The expression of ABH and Lewis antigens in Brazilian semi-isolated Black communities. Genet. Mol. Biol..

[43] Nordgren J, Nitiema LW, Ouermi D, Simpore J, Svensson L (2013). Host genetic factors affect susceptibility to norovirus infections in Burkina Faso. PloS One.

[44] Le Pendu J, Lemieux RU, Oriol R (1982). Purification of anti-Lec antibodies with specificity for beta DGal(1 replaced by 3)beta DGlcNAcO- using a synthetic immunoadsorbent. Vox Sang..

[45] Daniels, G. *Human Blood Groups*. (Wiley, 2013).

[46] Schnaar RL, Gerardy-Schahn R, Hildebrandt H (2014). Sialic acids in the brain: gangliosides and polysialic acid in nervous system development, stability, disease, and regeneration. Physiol. Rev..

[47] Delorme C (2001). Glycosphingolipid Binding Specificities of Rotavirus: Identification of a Sialic Acid-Binding Epitope. J. Virol..

[48] Muchmore EA, Diaz S, Varki A (1998). A structural difference between the cell surfaces of humans and the great apes. Am. J. Phys. Anthropol..

[49] Rougemont Ade (2016). Clinical severity and molecular characteristics of circulating and emerging rotaviruses in young children attending hospital emergency departments in France. Clin. Microbiol. Infect..

[50] Wilmore JR (2018). Commensal Microbes Induce Serum IgA Responses that Protect against Polymicrobial Sepsis. Cell Host Microbe.

[51] Rausch P (2011). Colonic mucosa-associated microbiota is influenced by an interaction of Crohn disease and FUT2 (Secretor) genotype. Proc. Natl. Acad. Sci. USA.

[52] Wacklin P (2011). Secretor genotype (FUT2 gene) is strongly associated with the composition of Bifidobacteria in the human intestine. PloS One.

[53] Tong M (2014). Reprograming of gut microbiome energy metabolism by the FUT2 Crohn’s disease risk polymorphism. ISME J..

[54] Turpin, W. *et al*. FUT2 genotype and secretory status are not associated with fecal microbial composition and inferred function in healthy subjects. *Gut Microbes* 1–12 10.1080/19490976.2018.1445956 (2018). 10.1080/19490976.2018.1445956 PMC6219652 29533703

[55] Davenport ER (2016). ABO antigen and secretor statuses are not associated with gut microbiota composition in 1,500 twins. BMC Genomics.

[56] Kazi AM (2017). Secretor and Salivary ABO Blood Group Antigen Status Predict Rotavirus Vaccine Take in Infants. J. Infect. Dis..

[57] Bucardo F (2018). The Lewis A phenotype is a restriction factor for Rotateq and Rotarix vaccine-take in Nicaraguan children. Sci. Rep..

[58] Blutt SE (2017). Gastrointestinal microphysiological systems. Exp. Biol. Med. Maywood NJ.

[59] Saxena K (2015). Human Intestinal Enteroids: a New Model To Study Human Rotavirus Infection, Host Restriction, and Pathophysiology. J. Virol..

[60] Rillahan CD (2012). Global Metabolic Inhibitors of Sialyl- and Fucosyltransferases. Nat. Chem. Biol..

[61] Marionneau S, Airaud F, Bovin NV, Le Pendu J, Ruvoën-Clouet N (2005). Influence of the combined ABO, FUT2, and FUT3 polymorphism on susceptibility to Norwalk virus attachment. J. Infect. Dis..

