# Supplementary material for: Histo-blood group antigen-binding specificities of human rotaviruses are associated with gastroenteritis but not with in vitro infection
Source: Sci Rep. 2018 Aug 28;8:12961. doi: 10.1038/s41598-018-31005-4 (PMC6113245; doi:10.1038/s41598-018-31005-4)
Supplement: Supplementary file 1 — Supplementary Information [file 41598_2018_31005_MOESM1_ESM.docx]

**Histo-blood group antigen-binding specificities of human rotaviruses are associated with gastroenteritis but not with *in vitro* infection**

Laure Barbé^1^, Béatrice Le Moullac-Vaidye^1^, Klara Echasserieau^1,2^, Karine Bernardeau^1,2^, Thomas Carton^3^, Nicolai Bovin^4^, Johan Nordgren^5^, Lennart Svensson^5,6^, Nathalie Ruvoën-Clouet^1,7^ and Jacques Le Pendu^1*^

^1^CRCINA, Inserm, Université d’Angers, Université de Nantes, Nantes, France

^2^Plateforme P2R « Production de protéines recombinantes », SFR Sante F. Bonamy-IRS-UN, Université de Nantes, INSERM, CNRS, CHU Nantes, Nantes, France

^3^Biofortis, Mérieux NutriSciences, Nantes, France

^4^Institute of Bioorganic Chemistry RAS, Moscow, Russia

^5^Division of Molecular Virology, Medical Faculty, University of Linköping, Linköping, Sweden

^6^Division of Infectious Diseases, Department of Medicine Solna, Karolinska Institute, Stockholm, Sweden

^7^Oniris, Ecole Nationale Vétérinaire, Agroalimentaire et de l’Alimentation, Nantes, France

* Correspondence to: jacques.le-pendu@univ-nantes.fr

**Supplementary Table S1. Short names and structures of neoglycoconjugates used in this study**

| **Short Name** | **Structure** |
| --- | --- |
| LacdiNAc | GalNAcβ4GlcNAcβ-R1 |
| Tk antigen | GlcNAcβ6[GlcNAcβ3]Galβ4GlcNAcβ-R1 |
| LacNAc | Galβ4GlcNAc-R1 |
| O-glycan core 5 | GalNAcα3GalNAcα-R1 |
| Tββ antigen | Galβ3GalNAcβ-R1 |
| Chitobiose | GlcNacβ4GlcNac-R1 |
| Tri LacNAc | Galβ4GlNAcβ3Galβ4GlNAcβ3Galβ4GlcNAcβ-R1 |
| H type 2 | Fucα2Galβ4GlcNAcβ-R1 |
| O-glycan core 3 | GlcNAcβ3GalNAcα-R1 |
| Le^c^ 3’-LacNAc | Galβ3GlcNAcβ3Galβ4GlcNAcβ-R1 |
| Le^c^ 6’-LacNAc | Galβ3GlcNAcβ6Galβ4GlcNAcβ-R1 |
| LacNAc3’-GlcNAc6’-LacNAc | Galβ4GlcNAcβ3[GlcNAcβ6]Galβ4GlcNAcβ-R1 |
| (LacNAc)_2_3’,6’-LacNAc | Galβ4GlcNAcβ3[Galβ4GlcNAcβ6]Galβ4GlcNAcβ-R1 |
| LacNAc3’-LacNAc | Galβ4GlcNAcβ3Galβ4GlcNacβ-R1 |
| LacNAc6’-LacNAc | Galβ4GlcNAcβ6Galβ4GlcNacβ-R1 |
| GlcNAc3’-LacNAc6’-LacNAc | GlcNAcβ3[Galβ4GlcNAcβ6]Galβ4GlcNAcβ-R1 |
| Lacto-N-Fucopentaose | Fucα2Galβ3GlcNAcβ3Galβ4Glcβ-R1 |
| A tri | GalNAcα3[Fucα2]Galβ-R1/R2 |
| A hexa | GalNAcα3[Fucα2]Galβ3GlcNAcβ3Galβ4Glcβ-R2 |
| A Lewis b hepta | GalNAcα3[Fucα2]Galβ3[Fucα4]GlcNAcβ3Galβ4Glcβ-R2 |

R1: spacer-PAA (polyacrylamide)

R2: spacer-HSA (human serum albumin)

**
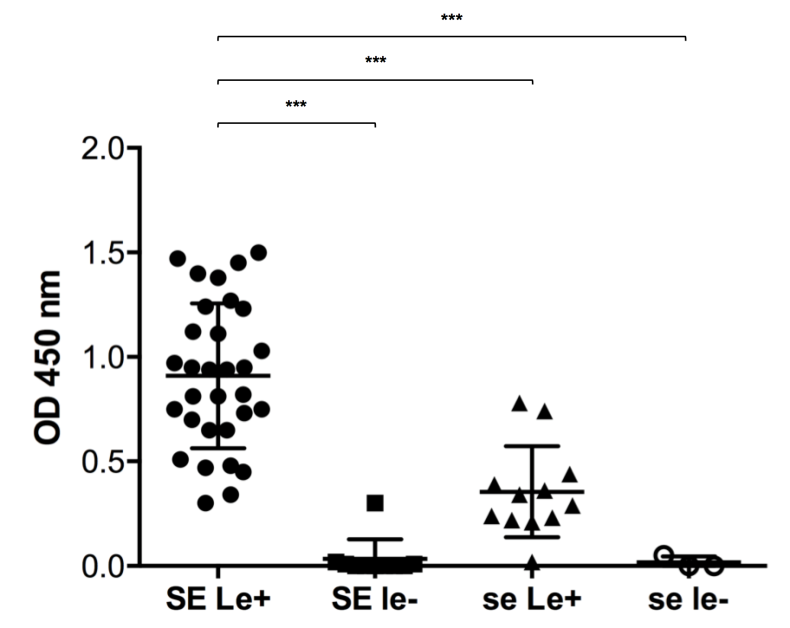
**

**Supplementary figure S1. The Lewis b antigen is detected in saliva from Secretor and Lewis positive individuals and not, or to much lower levels, in saliva from non-secretor and/or Lewis negative individuals.** Lewis b expression on a panel of previously well-defined secretor and Lewis types saliva samples (SE = Secretor (FUT2^+^); se = non-secretor (FUT2^-^); Le^+^ = Lewis positive (FUT3^+^); le^-^ = Lewis negative (FUT3^-^)) was tested by ELISA using the 2-25LE antibody (Thermo Fisher Scientific). The small reactivity observed with saliva of the se Le^+^ group is likely due to a cross-reactivity of the antibody with the Lewis a epitope (Bara J, Gautier R, Le Pendu J, Oriol R. 1988, Immunochemical characterization of mucins. Polypeptide (M1) and polysaccharide (A and Leb) antigens. *Biochem. J.*, 254, 185-193). Mann-Whitney test was used to compare groups (***p<0.001).

**
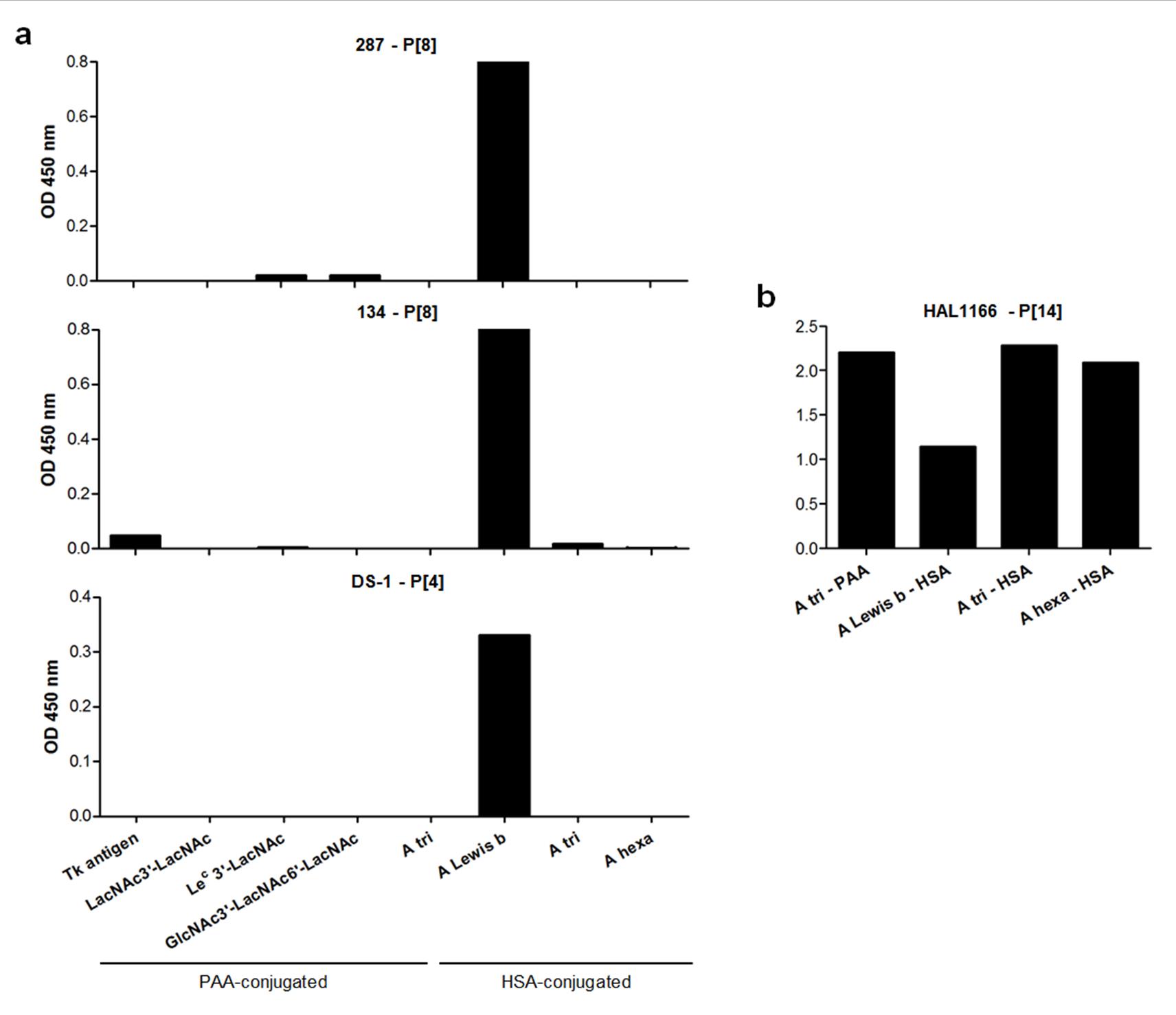
**

**Supplementary figure S2. Binding specificity of VP8* proteins to HBGAs measured by synthetic oligosaccharide-based binding assay.** VP8* proteins from the 287 and 134 clinical strains (P[8]), the cell culture-adapted strains DS-1 (P[4]) and HAL1166 (P[14]) were tested for binding to a panel of synthetic oligosaccharides (see Table S1 for structures). Background values around 0.05 OD units were subtracted from raw data. VP8* from P[8] and P[4] strains specifically recognize the A Lewis b antigen only **(a)**, whereas VP8* from the HAL1166 P[14] strain bind the A tri, A Lewis b hepta and A hexa antigens **(b)**.

**
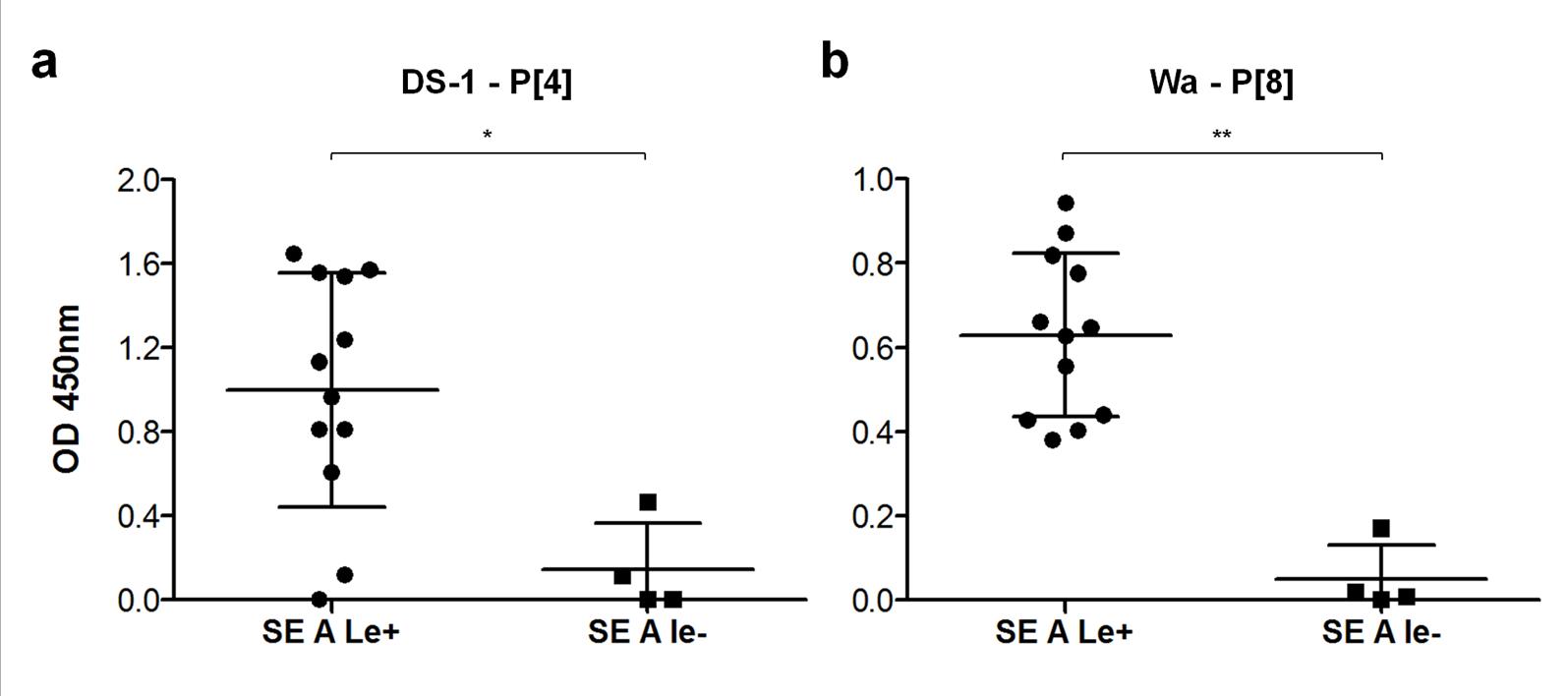
Supplementary figure S3. Binding of the DS-1 (P[4]) and Wa (P[8]) VP8* to HBGAs measured by saliva-based binding assay.** VP8* proteins from the cell culture-adapted strains DS-1 (P[4]) **(a)** and Wa (P[8]) **(b)** were tested for binding to a panel of previously well-defined A, secretor and Lewis types saliva samples (SE = Secretor (FUT2^+^); Le^+^ = Lewis positive (FUT3^+^); le^-^ = Lewis negative (FUT3^-^); A = Blood group A). The binding profile of these VP8* proteins to HBGAs is identical with a binding to mucins of SE/A/Le^+^ phenotypes and a much weaker recognition of SE/A/Le^-^ mucins (SE/A/Le^+^ n=12; SE/A/le^-^ n=4). Mann-Whitney test was used to compare groups (**p<0.01, *p<0.05).

**Supplementary figure S4. Absence of correlation between the binding of P[6] VP8* to Lewis negative saliva samples and H type 1 expression.** VP8* proteins from a representative P[6] clinical strain (225) isolated in Burkina Faso were tested for binding to a panel of Lewis negative saliva samples (SE = Secretor (FUT2^+^); se = non-secretor (FUT2^-^); le^-^ = Lewis negative (FUT3^-^)). H type 1 expression on these Lewis negative saliva samples was tested by ELISA using the BC2L-C Nter lectin or an anti-BG-4 monoclonal antibody (Covance). No association was found using Spearman’s correlation.

**
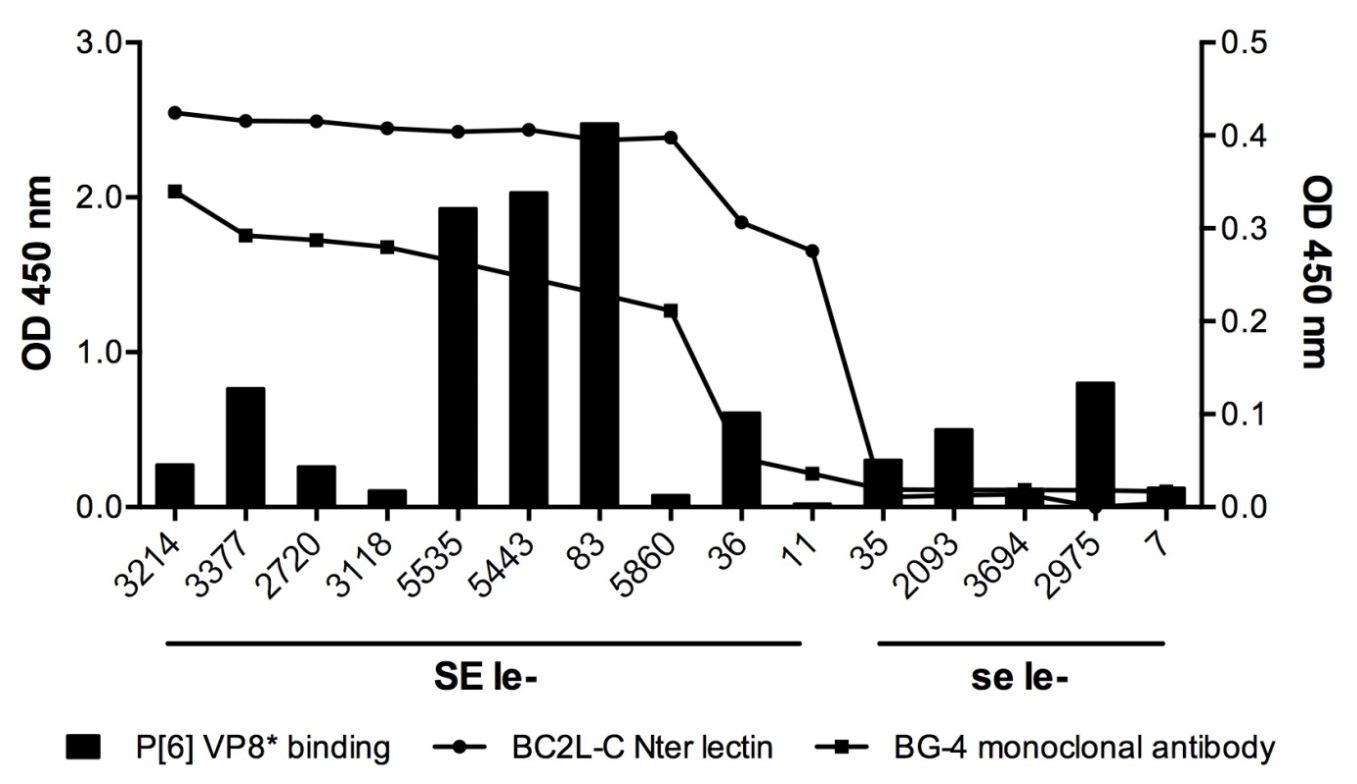
**

**Supplementary figure S5. Effect of sialidase treatment on binding of VP8* from SA11 RV (P[2]) to MA-104 cells.** Sialidase treatment efficacy was controlled by testing expression of α(2,6)-sialic acids using the *Sambucus nigra* agglutinin (SNA) **(a)** before analyzing its effect on SA11 VP8* binding **(b)** by flow cytometry: negative controls with secondary antibodies only (light grey); positive controls on buffer-treated cells (solid line); sialidase-treated cells (dotted line). The results provided are representative of those obtained from at least two independent experiments.

**
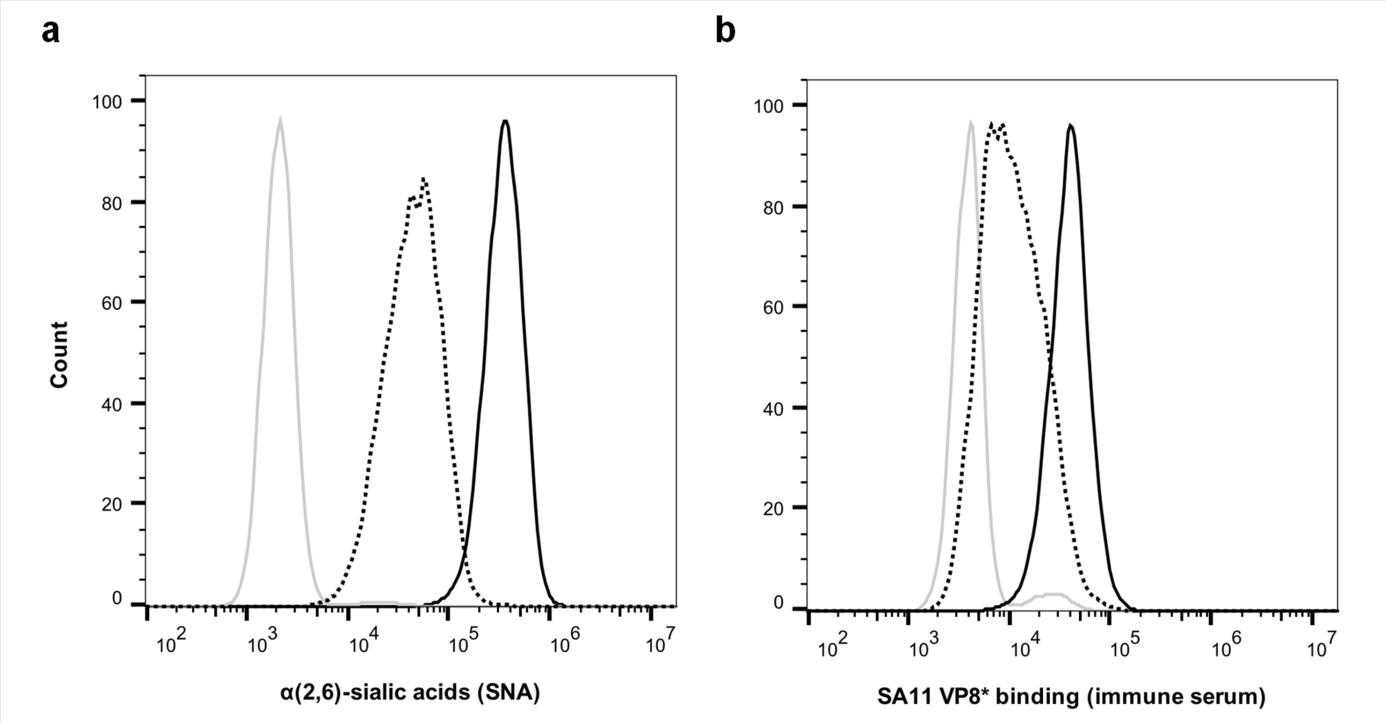
**

**
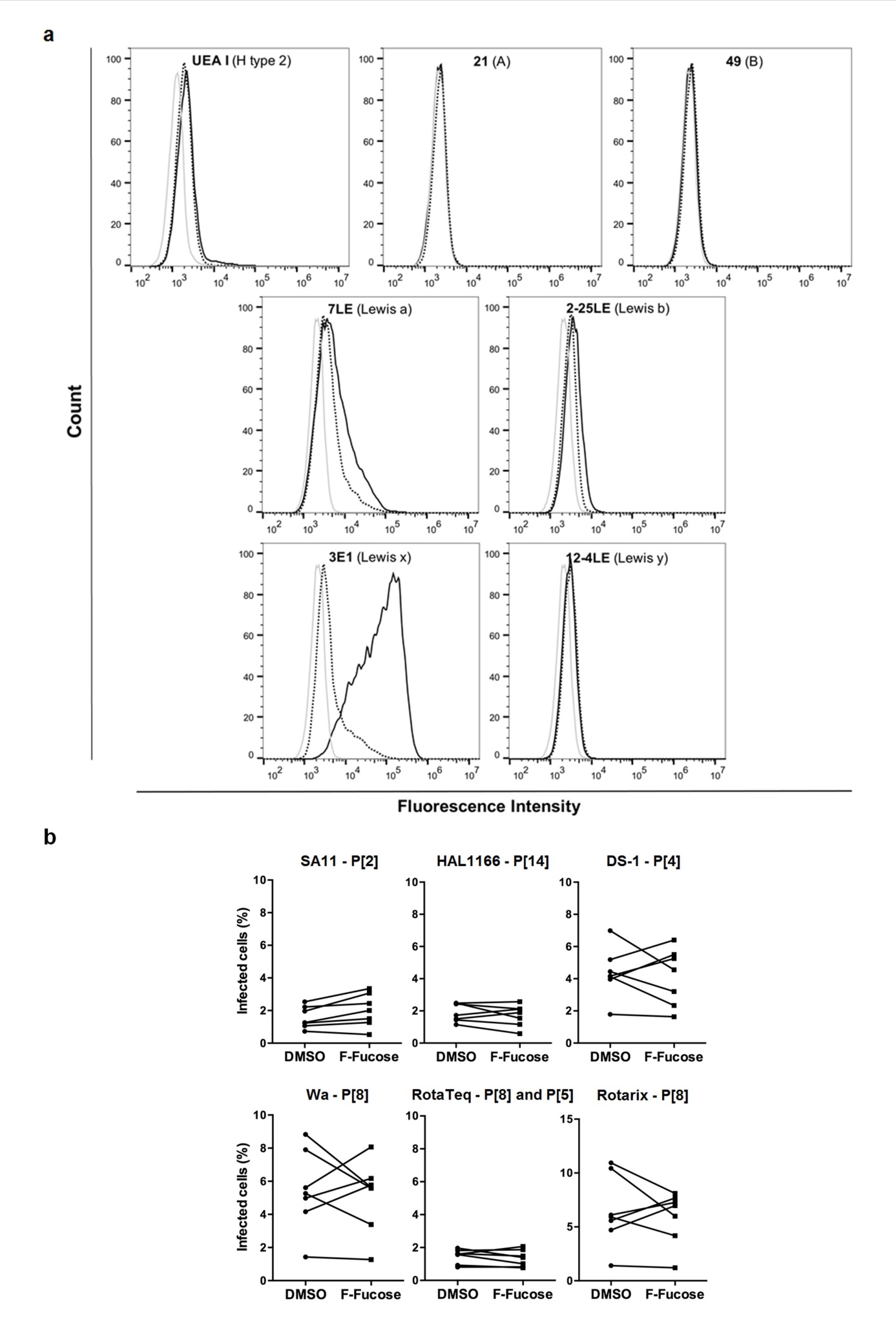
**

**Supplementary figure S6. Effect of fucose synthesis blockade on *in vitro* infection of Vero cells. (a)** 2F-fucose treatment efficacy was controlled by testing expression of various HBGAs (H type 2, A, B, Lewis a, Lewis b, Lewis x and Lewis y antigens) by flow cytometry: negative controls with secondary antibodies only (light grey); positive controls on DMSO treated cells (solid line); 2F-fucose treated cells (dotted line). The results provided are representative of those obtained from at least three independent experiments. **(b)** Infection of Vero cells, either DMSO (control) or 2F-fucose treated, by indicated cell culture-adapted strains of RV was quantified by fluorescence microscopy with an ArrayScan HCS Reader (Thermo Scientific). The results of each independent experiment are shown by linked control (DMSO) and treated (2F-fucose) values (n=7 for each strain of RV).

**
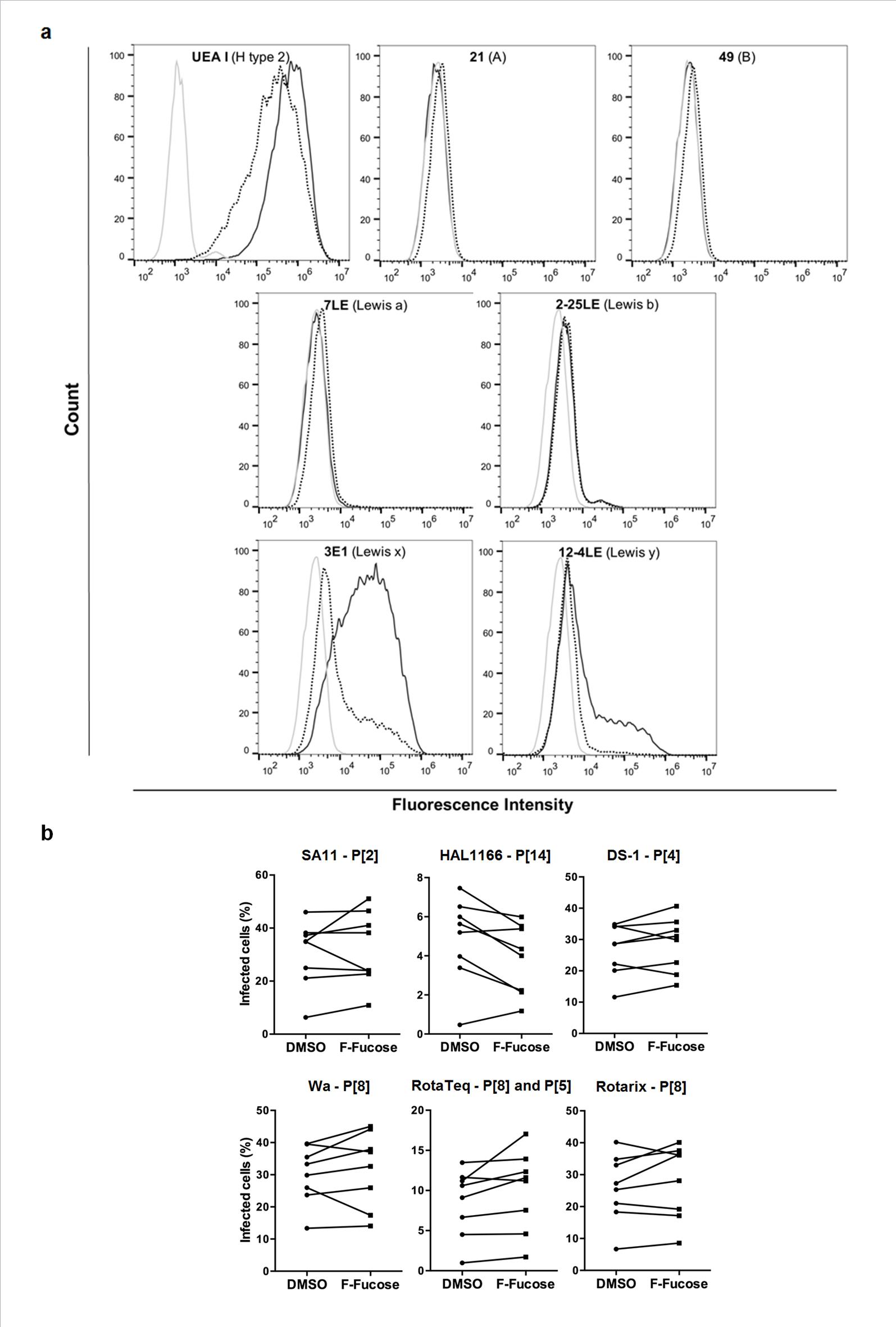
**

**Supplementary figure S7. Effect of fucose synthesis blockade on *in vitro* infection of Caco-2 cells. (a)** 2F-fucose treatment efficacy was controlled by testing expression of various HBGAs (H type 2, A, B, Lewis a, Lewis b, Lewis x and Lewis y antigens) by flow cytometry: negative controls with secondary antibodies only (light grey); positive controls on DMSO treated cells (solid line); 2F-fucose treated cells (dotted line). The results provided are representative of those obtained from at least three independent experiments. **(b)** Infection of Caco-2 cells, either DMSO (control) or 2F-fucose treated, by indicated cell culture-adapted strains of RV was quantified by fluorescence microscopy with an ArrayScan HCS Reader (Thermo Scientific). The results of each independent experiment are shown by linked control (DMSO) and treated (2F-fucose) values (n=8 for each strain of RV).

**
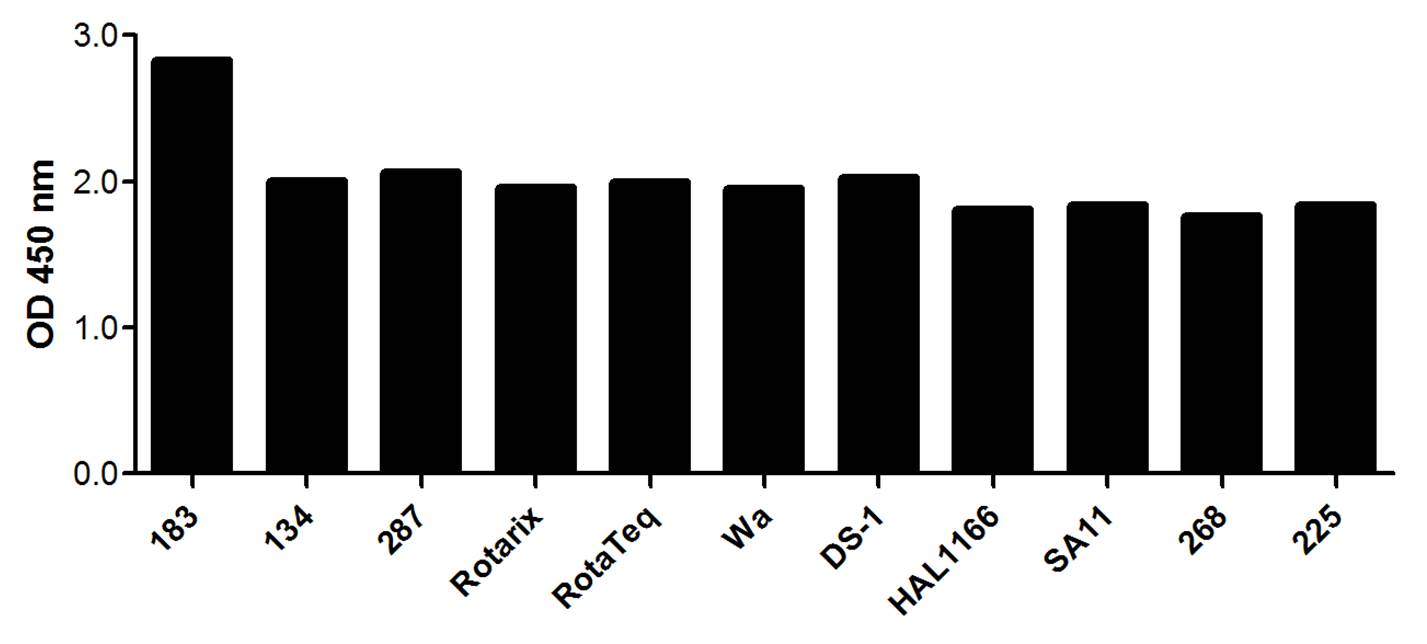
Supplementary figure S8. Recognition of the VP8* proteins used in this study by the rat anti-VP8* polyclonal antibody.** The antiserum recognition specificity for all the VP8* proteins studied was tested at the dilution used in the saliva- and oligosaccharide-based binding assays (1:500). The background value (0.07 OD units) was subtracted from raw data. Clinical strains: 183, 134 and 287 (P[8]); 268 and 225 (P[6]); cell culture-adapted strains: Wa (P[8]), DS-1 (P[4]), HAL1166 (P[14]) and SA11 (P[2]); vaccine strains: Rotarix and RotaTeq (P[8]).

**Supplementary Methods**

**Production of VP8* proteins**

The GenBank accession numbers for the VP8* sequences of Rotarix (P[8]); RotaTeq (P[8] strain); SA11 (P[2]); HAL1166 (P[14]); DS-1 (P[4]); Wa (P[8]); French isolates 134, 183, 287 (P[8]) and Burkinabe isolates 225 and 268 (P[6]) are the following: JN849113, GU565044.1, LC178567.1, L20875.1, EF672577.1, KT694942.1, MG649047, MG649048, MG649049, MG602259 and MG602260 respectively.

**Rat anti-VP8* polyclonal antibody generation**

The rat anti-VP8* polyclonal antibody was generated by serial inoculations of two rats with purified VP8* following GST cleavage. Recognition of target VP8* of the different P types was confirmed using ELISA (Fig. S8). Rat inoculations were performed at the animal experimentation core facility of the University of Nantes (IRS-UN facility agreement #C44278) and were approved by the national ethics review board of the French Ministry of Enseignement Supérieur et de la Recherche (project licence #02375.02). The animal care and use protocol adhered to European Directive number 2010/63.

**Saliva samples**

Saliva samples collected from 59 healthy individuals of known ABO, Secretor and Lewis phenotypes, with confirmed *FUT2* (Secretor) and *FUT3* (Lewis) genotypes as previously described were selected to cover the ABO, Secretor and Lewis phenotypic diversity. The ABO, Secretor and Lewis phenotypes and genotypes were obtained as previously described (Azevedo, M., Eriksson, S., Mendes, N., Serpa, J., Figueiredo, C., Resende, L. P., Ruvoen-Clouet, N., Haas, R., Boren, T., Le Pendu, J., and David, L. Infection by Helicobacter pylori expressing the BabA adhesin is influenced by the secretor phenotype. *J. Pathol.* **215**, 308-316 (2008) ; Marionneau, S., Airaud, F., Bovin, N. V., Le Pendu, J., and Ruvoen-Clouet, N. Influence of the combined ABO, FUT2, and FUT3 polymorphism on susceptibility to Norwalk virus attachment. *J. Infect. Dis.* **192,** 1071–1077 (2005)). After collection, samples were boiled for 10 minutes and centrifuged for 5 minutes at 13,000 g. The clear supernatant was stored at -20°C until use. Saliva samples collection was approved by the Nantes University Hospital Review Board (study no. BRD02/2-P), and informed consent was obtained from all saliva donors.

**H type 1 and Lewis b phenotyping by ELISA**

Expression of H type 1 and Lewis b on saliva samples was tested by ELISA according to the major steps described in the main methods. The reagents used to detect H type 1 were the biotinylated BC2L-C Nter lectin at 1:400 and an anti-BG-4 monoclonal antibody (Covance) at 1:10 followed by HRP-conjugated streptavidin at 1:500 or HRP-conjugated goat anti-mouse IgG (Uptima) at 1:1,000 respectively. For the detection of Lewis b, the 2-25LE antibody (Thermo Fisher Scientific) was used at 1:400 followed by HRP-conjugated goat anti-mouse IgG (Uptima) at 1:1,000.

**Cells and viruses**

The human RV strains HAL1166 and DS-1 as well as the simian strain SA11 were obtained from S. Le Guyader, IFREMER, Nantes, France. The human strain Wa was obtained from D. Poncet, CNRS, Gif-sur-Yvette, France. All strains were propagated in MA-104 cells in absence of Fetal Bovine Serum (FBS). MA-104 cell line was grown in Medium 199 (M199) and Caco-2, HT-29 and Vero cell lines were grown in Dulbecco’s Modified Eagle’s Medium (DMEM). Each medium was supplemented with 10% FBS, 2 mM of L-glutamine, 100 U/ml of penicillin and 100 µg/ml of streptomycin. DMEM was also supplemented with 1% of Non-Essentiel Amino Acids (NEAA) solution for the Caco-2 cell line culture, which was performed in 90% (vol/vol) air with 10% (vol/vol) CO_2_ instead of 95% (vol/vol) air with 5% (vol/vol) CO_2_ for the other cells. All RV strains were semi-purified using ultracentrifugation. RV-infected MA-104 cell cultures were harvested. After three cycles of freeze/thawing and clarification by a low-speed centrifugation, the viruses in the supernatants were concentrated by pelleting them at 100,000 g for 1.5 h through a 35% sucrose cushion using a SW32 Ti rotor (Beckman). The viruses in the pellets were resuspended in TNC buffer (20 mM Tris-HCl, 100 mM NaCl, 1 mM CaCl2, pH 8.0).

**Flow cytometry phenotyping**

The presence of histo-blood group A, B, Lewis a, Lewis b, Lewis x and Lewis y antigens at the cell surface was detected using primary antibodies 9113D10 (Diagast laboratories, France), 9621A8 (Diagast laboratories, France), 7LE (Thermo Fisher Scientific), 2-25LE (Thermo Fisher Scientific), 3E1 (obtained from Dr. D. Blanchard, EFS, Nantes, France) and 12-4LE (obtained from Dr. J. Bara, CNRS, Villejuif, France), respectively, and with FITC-labeled anti-mouse IgG (H+L) antibody (Beckman Coulter) as secondary reagent. The presence of histo-blood group H antigen and α(2,6)-linked sialic acids was detected with biotinylated UEA-I lectin (Vector laboratories) and SNA lectin (Vector laboratories) respectively, followed by PE-labeled streptavidin (BD Biosciences). For sialidase treatment, cells were resuspended in medium at pH 5.5 in the presence or absence of 500 units of α(2-3,6,8)-sialidase (New England BioLabs) and incubated for 4 h at 37°C. Cells were washed, reacted with GST-VP8* or SNA lectin, and stained as described in the main methods.
